# Supplementary material for: Single-Cell Gene Network Analysis and Transcriptional Landscape of MYCN-Amplified Neuroblastoma Cell Lines
Source: Biomolecules. 2021 Jan 28;11(2):177. doi: 10.3390/biom11020177 (PMC7912277; doi:10.3390/biom11020177)
Supplement: Supplementary file 1 [file biomolecules-11-00177-s001.zip › SuppFile_S3_rmarkdown.html]

Single-Cell Gene Network Analysis and Transcriptional Landscape of MYCN-amplified Neuroblastoma cell lines


# Single-Cell Gene Network Analysis and Transcriptional Landscape of MYCN-amplified Neuroblastoma cell lines

#### Federico M. Giorgi

#### 21 January 2021

- 1 Extract gene count matrices
- 2 Setting up the R environment for the analysis
- 3 Prepare data
  - 3.1 Load raw counts data and convert gene ids to gene symbols
  - 3.2 Normalization
- 4 Descriptive plots
  - 4.1 Expression by chromosome band
- 5 Comparison with bulk RNA-Seq data
  - 5.1 Loading Harenza bulk-seq data
  - 5.2 Comparison between single cell and bulk RNA-Seq
- 6 Clustering single cell data
  - 6.1 Cell Cycle clustering
  - 6.2 Read number clustering
- 7 Differential expression analysis
  - 7.1 Comparison with bulk signature
  - 7.2 Visualization of differential expression by volcano plot.
- 8 Pathway enrichment analysis
  - 8.1 Whole-signature GSEA
  - 8.2 Single-cell GSEA
- 9 Master Regulator Analysis
- 10 Single Cell Master Regulator Analysis
- 11 Louvain Clustering
  - 11.1 Assign cells to clusters
    - 11.1.1 Clustering at higher resolution (0.2)
- 12 Dissection of heterogeneity
- 13 Technical Session Info

# 1 Extract gene count matrices

Gene counts were extracted from 10X genomics H5 matrices using cellranger v4.0.0 on Linux Ubuntu 18.04. This yielded comma-separated value files (CSV) with genes as rows, samples (cells) as columns, and values corresponding to the raw read counts observed for every gene/cell combination. The CSV files (one per cell type) are then zipped.

```
# Linux code
sample="Kelly"
cellranger mat2csv ${sample}/filtered_gene_bc_matrices_h5.h5 ${sample}.csv
sample="BE2C"
cellranger mat2csv ${sample}/filtered_gene_bc_matrices_h5.h5 ${sample}.csv
gzip *csv
```

# 2 Setting up the R environment for the analysis

We will load all the packages required by the full analysis, plus extra functions available in the “code/” subdirectory. If the packages are not available, we will install them using Bioconductor.

```
if (!requireNamespace("BiocManager",quietly=TRUE)){
  install.packages("BiocManager")
}
packages<-c("AnnotationDbi","biomaRt","corto","DESeq2",
            "dplyr","ggplot2","grid","gridExtra",
            "msigdbr","org.Hs.eg.db","org.Mm.eg.db",
            "pander","Rtsne","Seurat","slalom",
            "stringr","TeachingDemos",
            "tidyverse","xlsx"
)
for(p in packages){
  if (!p %in% rownames(installed.packages())){
    BiocManager::install(p)
  }
  library(p,character.only=TRUE)
}
source("code/area.R") # Functions to perform advanced rank enrichment analysis (area) for single cell GSEA
source("code/geneids.R") # Functions to convert gene ids
source("code/heatmaps.R") # Functions to plot Heatmaps
source("code/expmerger.R") # Functions to sum multiple expression tracks
source("code/qol.R") # Various "quality of life" functions
source("code/vst.R") # Variance Stabilizing Transformation functions
```

# 3 Prepare data

Colors associated to cell lines are defined here

```
col_be2c<-"salmon"
col_kelly<-"cornflowerblue"
```

## 3.1 Load raw counts data and convert gene ids to gene symbols

```
if(!file.exists("data/rawcounts_symbols.rda")){
  ### Load counts (from CellRanger) ----
  rawcounts_be2c<-read.csv(gzfile("data/BE2C.csv.gz"),as.is=TRUE,row.names=1)
  ncol(rawcounts_be2c) # 962 BE2C cells
  rawcounts_kelly<-read.csv(gzfile("data/Kelly.csv.gz"),as.is=TRUE,row.names=1)
  ncol(rawcounts_kelly) # 1105 Kelly cells
  # R loads "-" as ".", we must fix this
  colnames(rawcounts_be2c)<-gsub("\\.","-",colnames(rawcounts_be2c))
  colnames(rawcounts_be2c)<-paste0("be2c_",colnames(rawcounts_be2c))
  colnames(rawcounts_kelly)<-gsub("\\.","-",colnames(rawcounts_kelly))
  colnames(rawcounts_kelly)<-paste0("kelly_",colnames(rawcounts_kelly))
  # Merge the two datasets in a single raw counts matrix
  # rows are genes, columns are single cells
  rawcounts<-cbind(rawcounts_be2c,rawcounts_kelly)
  rawcounts<-as.matrix(rawcounts)
  dim(rawcounts) # 32738 ENSEMBL genes, 2067 samples
  #save(rawcounts,file="data/rawcounts_ensg.rda")
  
  ## Convert to gene symbols
  # Ensembl ids mapping to the same gene will see their raw counts summed up
  ensgmat<-rawcounts
  tmp<-ens2eg(rownames(ensgmat))
  convlist<-eg2sym(tmp)
  names(convlist)<-names(tmp)
  rawcounts<-squish(ensgmat,convlist=convlist,method="sum",verbose=TRUE)
  dim(rawcounts) # 22135 genes, 2067 samples
  save(rawcounts,file="data/rawcounts_symbols.rda")
  
  ## Save as CSV
  ksym<-rawcounts[,grep("kelly",colnames(rawcounts))]
  bsym<-rawcounts[,grep("be2c",colnames(rawcounts))]
  write.csv(bsym,file="results/Supp_File_S1_BE2C_rawcounts.csv")
  write.csv(ksym,file="results/Supp_File_S2_Kelly_rawcounts.csv")
} else {load("data/rawcounts_symbols.rda")}
```

## 3.2 Normalization

Normalization of raw count data will be performed with the FPKM and TPM algorithms, with DESeq2-VST (Variance Stabilizing Transformation) and with the LogNormalize function of Seurat.

```
## Normalization
# Extract gene lengths (more precisely, transcript lengths)
fname<-"data/genelengths.rda"
if(!file.exists(fname)){
  library(GenomicFeatures)
  supportedUCSCtables(genome="hg38", url="http://genome.ucsc.edu/cgi-bin/")
  hg<-makeTxDbFromUCSC(genome="hg38",tablename="refGene")
  exonic<-exonsBy(hg,by="gene")
  redexonic<-reduce(exonic)
  genelengths<-sum(width(redexonic))
  names(genelengths)<-eg2sym(names(genelengths))
  genelengths<-genelengths[!duplicated(names(genelengths))]
  genelengths<-genelengths[genelengths>0]
  genelengths<-genelengths[!is.na(genelengths)]
  save(genelengths,file=fname)
}else{load(fname)}

# Function to calculate FPKM (https://www.rna-seqblog.com/rpkm-fpkm-and-tpm-clearly-explained/)
fpkm<-function(counts,genelengths){
  common<-intersect(rownames(counts),names(genelengths))
  counts<-counts[common,]
  lengths<-genelengths[common]
  fpms<-apply(counts,2,function(x){1E6*x/sum(x)})
  fpkms<-fpms
  for(i in 1:nrow(fpms)){
    fpkms[i,]<-1E3*fpms[i,]/lengths[i]
  }
  return(fpkms)
}
fname<-"data/fpkms.rda"
if(!file.exists(fname)){
  fpkms<-fpkm(rawcounts,genelengths)
  save(fpkms,file=fname)
}
# Function to calculate TPM (https://www.rna-seqblog.com/rpkm-fpkm-and-tpm-clearly-explained/)
tpm<-function(counts,genelengths){
  common<-intersect(rownames(counts),names(genelengths))
  counts<-counts[common,]
  lengths<-genelengths[common]
  intermediate<-counts
  for(i in 1:nrow(counts)){
    intermediate[i,]<-1E3*counts[i,]/lengths[i]
  }
  tpms<-apply(intermediate,2,function(x){1E6*x/sum(x)})
  return(tpms)
}
fname<-"data/tpms.rda"
if(!file.exists(fname)){
  tpms<-tpm(rawcounts,genelengths)
  save(tpms,file=fname)
}

# Variance-Stabilizing Transformation (expmat)
fname<-"data/tpms.rda"
if(!file.exists(fname)){
  vstmat<-vst(rawcounts)
  save(vstmat,file=fname)
}

## Log-Normalize with Seurat
# By default, Seurat employs a global-scaling normalization method LogNormalize that
# normalizes the gene expression measurements for each cell by the total expression,
# multiplies this by a scale factor (10,000 by default), and log-transforms the result:
fname<-"data/expmat.rda"
if(!file.exists(fname)){
  seuset<-CreateSeuratObject(counts=rawcounts,project="nblcells",min.cells=3,min.features=1000)
  seuset # 15782 genes, 2067 samples
  seuset<-NormalizeData(seuset,normalization.method="LogNormalize",scale.factor=10000)
  save(seuset,file="data/seuset.rda")
  
  ## Mean variability plot showing most expressed and variable genes
  expmat<-as.matrix(seuset[["RNA"]]@data)
  dim(expmat) # 15782 genes, 2067 samples
  save(expmat,file=fname)
}
```

# 4 Descriptive plots

```
### Descriptive plots using TPMs ----
load("data/tpms.rda")
fname<-"results/tpms.csv"
if(!file.exists("results/tpms.csv.gz")){
  if(!file.exists(fname)){
    write.csv(tpms,file=fname)
  }}
bmat<-tpms[,grep("be2c",colnames(tpms))]
kmat<-tpms[,grep("kelly",colnames(tpms))]


### Variance vs. Expression ----
bexpmean<-apply(bmat,1,mean)
kexpmean<-apply(kmat,1,mean)
bexpvar<-apply(bmat,1,var)
kexpvar<-apply(kmat,1,var)


# Define pseudovalues for log10 transform
min(kexpmean[kexpmean!=0]) # 0.0006598291
```

```
## [1] 0.0006598291
```

```
min(bexpmean[bexpmean!=0]) # 0.0003172931
```

```
## [1] 0.0003172931
```

```
pseudo<-0.0001
min(kexpvar[kexpvar!=0]) # 0.0003762165
```

```
## [1] 0.0003762165
```

```
min(bexpvar[bexpvar!=0]) # 0.00009684925
```

```
## [1] 9.684925e-05
```

```
pseudov<-0.0001


# Calculate fitted variance
bx<-log10(bexpmean+pseudo)
by<-log10(bexpvar+pseudov)
bres<-loess(formula=by~bx)$resid
kx<-log10(kexpmean+pseudo)
ky<-log10(kexpvar+pseudov)
kres<-loess(formula=ky~kx)$resid 


### Genes to show ----
## Housekeeping
toshow<-c("ACTB","GAPDH","B2M","GUSB")
## MYCN network
# Moreover, as existing literature refers to PRDM8, MYBL8, HMGB2 and TEAD4
# as crucial factors in the regulatory network triggered by MYCN, we examined how
# their transcription level varied across the two divergent neuroblastoma cell groups
# as a function of MYCN expression.
toshow<-c(toshow,c("MYCN","PRDM8","MYBL2","HMGB2","TEAD4"))
## Highly variable
bhighvar<-names(sort(bres,dec=TRUE))[1:4]
khighvar<-names(sort(kres,dec=TRUE))[1:4]
toshow<-c(toshow,khighvar,bhighvar)
## Low variance, high expression (candidate housekeeping)
bhk<-intersect(names(sort(bres,dec=FALSE))[1:500],names(sort(bx,dec=TRUE))[1:1000])
khk<-intersect(names(sort(kres,dec=FALSE))[1:200],names(sort(kx,dec=TRUE))[1:1000])
toshow<-c(toshow,bhk,khk)
## Other genes
toshow<-c(toshow,"MYC","MYCL","ALK","LMO1")
toshow<-unique(toshow)
```

```
# Variance vs. Expression plots
#png("plots/003_var_vs_exp.png",w=3000,h=3000,res=300)
set.seed(3)
par(mfrow=c(2,2))
scatter2(bx,by,main="BE2C",xlab="Log10 Average Expression (TPM)",ylab="Log10 Variance",col=col_be2c)
scatter2(bx,bres,main="BE2C",xlab="Log10 Average Expression (TPM)",ylab="Log10 Variance (Loess Residuals)",col=col_be2c,showLine=FALSE,extendXlim=TRUE)
textplot3(bx[toshow],bres[toshow],words=toshow,font=2)

scatter2(kx,ky,main="Kelly",xlab="Log10 Average Expression (TPM)",ylab="Log10 Variance",col=col_kelly)
scatter2(kx,kres,main="Kelly",xlab="Log10 Average Expression (TPM)",ylab="Log10 Variance (Loess Residuals)",col=col_kelly,showLine=FALSE,extendXlim=TRUE)
textplot3(kx[toshow],kres[toshow],words=toshow,font=2)
```

Figure S1. Variance vs. Expression plot

```
#dev.off()
```

```
### TPM vs. TPM ----
# Plot and compare genes in common (they should be the same already, but better to be sure)
#png("plots/003_tpm_vs_tpm_exp.png",w=4000,h=3000,res=600)
set.seed(6)
common<-intersect(names(bexpmean),names(kexpmean))
corto::scatter(bx,kx,xlab="BE2C Log10 Average Expression (TPM)",ylab="Kelly Log10 Average Expression (TPM)",main="Average Expression",col="gainsboro",extendXlim=TRUE)
textplot3(bx[toshow],kx[toshow],words=toshow,font=2)
```

Figure S2. TPM vs TPM Expression plot

```
#dev.off()
```

```
#png("plots/003_tpm_vs_tpm_var.png",w=4000,h=3000,res=600)
set.seed(6)
corto::scatter(bres,kres,xlab="BE2C Log10 Variance",ylab="Kelly Log10 Variance",main="Variance (Loess Residuals)",col="gainsboro",extendXlim=TRUE)
textplot3(bres[toshow],kres[toshow],words=toshow,font=2)
```

Figure S3. TPM vs TPM Variance plot

```
#dev.off()
```

```
### Housekeeping and MYCN expression ----
genes<-c("ACTB","GAPDH","B2M","GUSB")
#png("plots/003_exp_vs_mycn.png",w=4000,h=2500,res=300)
par(mfrow=c(2,4))
for(gene in genes){
  corto::scatter(log10(bmat["MYCN",]+pseudo),log10(bmat[gene,]+pseudo),main="BE2C",col=col_be2c,xlab="MYCN",ylab=gene)
}
for(gene in genes){
  corto::scatter(log10(kmat["MYCN",]+pseudo),log10(kmat[gene,]+pseudo),main="Kelly",col=col_kelly,xlab="MYCN",ylab=gene)
}
```

Figure S4. Housekeeping and MYCN expression

```
#dev.off()
```

```
### Expression vs. Cells with Gene ----
# Nr. cells with gene > 0
bcells<-apply(bmat,1,function(x){sum(x>0)})
kcells<-apply(kmat,1,function(x){sum(x>0)})

#png("plots/003_nrcells_be2c.png",w=4000,h=3000,res=600)
set.seed(2)
plot(bcells,bx,pch=20,col=col_be2c,xlim=c(-200,1400),xlab="Nr. Cells with Gene > 0 TPM",ylab="Log10 Average Expression (TPM)",main="BE2C")
mtext(paste0("Total: ",ncol(bmat)," cells"),cex=1,font=2)
textplot3(bcells[toshow],bx[toshow],words=toshow,font=2)
```

Figure S5. Number of cells in BE2C

```
#dev.off()
```

```
#png("plots/003_nrcells_kelly.png",w=4000,h=3000,res=600)
set.seed(2)
plot(kcells,kx,pch=20,col=col_kelly,xlim=c(-200,1400),xlab="Nr. Cells with Gene > 0 TPM",ylab="Log10 Average Expression (TPM)",main="Kelly")
textplot3(kcells[toshow],kx[toshow],words=toshow,font=2)
mtext(paste0("Total: ",ncol(kmat)," cells"),cex=1,font=2)
```

Figure S6. Number of cells in Kelly

```
#dev.off()
```

```
### Selected genes with error bars
error.bar <- function(x, y, upper,length=0.1,...){
  arrows(x,y+abs(upper), x, y, angle=90, code=3, length=length, ...)
}
# BE2C
#png("plots/003_somegenes_be2c.png",w=4000,h=3000,res=600)
par(las=2)
value<-sort(bx[toshow],dec=TRUE)+4
error<-bres[toshow]
bp<-barplot(value,main="BE2C",col=col_be2c,ylab=c("Log10 Average Expression (TPM)"),ylim=c(0,9),yaxt="n")
axis(2,at=c(0:9),labels=c(-4:5))
error.bar(bp[,1],value,error)
```

Figure S7. Selected genes with error bars in BE2C cells

```
#dev.off()
```

```
# Kelly
#png("plots/003_somegenes_kelly.png",w=4000,h=3000,res=600)
par(las=2)
value<-sort(kx[toshow],dec=TRUE)+4
error<-kres[toshow]
bp<-barplot(value,main="Kelly",col=col_kelly,ylab=c("Log10 Average Expression (TPM)"),ylim=c(0,9),yaxt="n")
axis(2,at=c(0:9),labels=c(-4:5))
error.bar(bp[,1],value,error)
```

Figure S8. Selected genes with error bars in Kelly cells

```
#dev.off()
```

## 4.1 Expression by chromosome band

These plots, highlighting the average TPM expression by chromosome band, are too wide to be printed in the Rmarkdown, and will therefore be printed in the plots/ folder.

```
### Chromosome bands ----
fname<-"data/mlist.rda"
if(!file.exists(fname)){
  mdf<-msigdbr(species="Homo sapiens") # Retrieve all human gene sets
  mlist<-mdf %>% split(x=.$gene_symbol,f=.$gs_name)
  save(mlist,file=fname)
}else{load(fname)}
chrom_bands <- mlist[grep("chr",names(mlist))] 
chrs<-c("chr1","chr2","chr3","chr4","chr5","chr6","chr7","chr8","chr9","chr10","chr11","chr12","chr13",
        "chr14","chr15","chr16","chr17","chr18","chr19","chr20","chr21","chr22","chrX","chrY")

# Find gene
gene<-"MYCN"
for(i in 1:length(chrom_bands)){
  band<-names(chrom_bands)[i]
  genes_here<-chrom_bands[[band]]
  if(gene%in%genes_here){
    message(gene," is in ",band)
  }
}
# MYCN is in chr2p24


# Kelly
cols<-c()
pickcols<-c("royalblue4","skyblue")
means<-c()
coords<-c()
for(i in 1:length(chrs)){
  chr<-chrs[i]
  herecol<-pickcols[(i%%2)+1]
  sub<-chrom_bands[grep(paste0(chr,"(p|q)"),names(chrom_bands))]
  herecols<-rep(herecol,length(sub))
  cols<-c(cols,herecols)
  coords<-c(coords,rep(chr,length(sub)))
  for(band in names(sub)){
    genes_here<-sub[[band]]
    genes_here<-intersect(rownames(kmat),genes_here)
    allexp<-kmat[genes_here,]
    mean<-mean(allexp)
    means<-c(means,mean)
    names(means)[length(means)]<-band
  }
}
```

```
png("plots/003_chrom_kelly.png",w=11000,h=1000,res=300)
par(las=2)
bp<-barplot(means,col=cols,ylab="Mean expression in band (TPM)",xaxt="n",main="Kelly gene expression by chromosome band",ylim=c(0,650))
for(i in 1:length(chrs)){
  chr<-chrs[i]
  xwhere<-mean(bp[which(coords==chr)])
  text(xwhere,600,labels=chr,font=2)
}
axis(1,at=bp,labels=names(means),cex.axis=0.7)
dev.off()
```

```
## png 
##   2
```

```
# BE2C
cols<-c()
pickcols<-c("red3","salmon")
means<-c()
coords<-c()
for(i in 1:length(chrs)){
  chr<-chrs[i]
  herecol<-pickcols[(i%%2)+1]
  sub<-chrom_bands[grep(paste0(chr,"(p|q)"),names(chrom_bands))]
  herecols<-rep(herecol,length(sub))
  cols<-c(cols,herecols)
  coords<-c(coords,rep(chr,length(sub)))
  for(band in names(sub)){
    genes_here<-sub[[band]]
    genes_here<-intersect(rownames(bmat),genes_here)
    allexp<-bmat[genes_here,]
    mean<-mean(allexp)
    means<-c(means,mean)
    names(means)[length(means)]<-band
  }
}
```

```
png("plots/003_chrom_be2c.png",w=11000,h=1000,res=300)
par(las=2)
bp<-barplot(means,col=cols,ylab="Mean expression in band (TPM)",xaxt="n",main="BE2C gene expression by chromosome band",ylim=c(0,650))
for(i in 1:length(chrs)){
  chr<-chrs[i]
  xwhere<-mean(bp[which(coords==chr)])
  text(xwhere,600,labels=chr,font=2)
}
axis(1,at=bp,labels=names(means),cex.axis=0.7)
dev.off()
```

```
## png 
##   2
```

# 5 Comparison with bulk RNA-Seq data

We will now compare neuroblastoma single cell RNA-Seq with bulk RNA-Seq data from a published dataset.

## 5.1 Loading Harenza bulk-seq data

Data was obtained from Harenza et al. (https://www.nature.com/articles/sdata201733). Here, we will load, format, and normalize them.

```
fname<-"data/harenza/rawcounts_symbols.rda"
if(!file.exists(fname)){
  ### Load counts (from CellRanger) ----
  rawcounts<-read.delim("data/harenza/harenza.counts.txt.gz",as.is=TRUE,skip=1,row.names=1)
  rawcounts<-as.matrix(rawcounts[,6:ncol(rawcounts)])
  colnames(rawcounts)<-gsub("\\.sorted\\.bam","",colnames(rawcounts))
  colnames(rawcounts)<-gsub("harenza_","",colnames(rawcounts))
  colnames(rawcounts)<-gsub("_.+","",colnames(rawcounts))
  colnames(rawcounts)<-gsub("\\.","-",colnames(rawcounts))
  save(rawcounts,file="data/harenza/rawcounts_ensg.rda")
  dim(rawcounts) # 58721 genes, 40 samples
  ## Convert to gene symbols
  ensgmat<-rawcounts
  rownames(ensgmat)<-gsub("\\..+","",rownames(ensgmat))
  tmp<-ens2eg(rownames(ensgmat))
  convlist<-eg2sym(tmp)
  names(convlist)<-names(tmp)
  rawcounts<-squish(ensgmat,convlist=convlist,method="sum",verbose=TRUE)
  dim(rawcounts) # 26131 genes, 40 samples
  save(rawcounts,file=fname)
} else {load(fname)}


### Normalization ----
# Extract gene lengths (more precisely, transcript lengths)
fname<-"data/genelengths.rda"
if(!file.exists(fname)){
  library(GenomicFeatures)
  supportedUCSCtables(genome="hg38", url="http://genome.ucsc.edu/cgi-bin/")
  hg<-makeTxDbFromUCSC(genome="hg38",tablename="refGene")
  exonic<-exonsBy(hg,by="gene")
  redexonic<-reduce(exonic)
  genelengths<-sum(width(redexonic))
  names(genelengths)<-eg2sym(names(genelengths))
  genelengths<-genelengths[!duplicated(names(genelengths))]
  genelengths<-genelengths[genelengths>0]
  genelengths<-genelengths[!is.na(genelengths)]
  save(genelengths,file=fname)
}else{load(fname)}


# Function to calculate FPKM (https://www.rna-seqblog.com/rpkm-fpkm-and-tpm-clearly-explained/)
fpkm<-function(counts,genelengths){
  common<-intersect(rownames(counts),names(genelengths))
  counts<-counts[common,]
  lengths<-genelengths[common]
  fpms<-apply(counts,2,function(x){1E6*x/sum(x)})
  fpkms<-fpms
  for(i in 1:nrow(fpms)){
    fpkms[i,]<-1E3*fpms[i,]/lengths[i]
  }
  return(fpkms)
}
fname<-"data/harenza/fpkms.rda"
if(!file.exists(fname)){
  fpkms<-fpkm(rawcounts,genelengths)
  save(fpkms,file=fname)
}
# Function to calculate TPM (https://www.rna-seqblog.com/rpkm-fpkm-and-tpm-clearly-explained/)
tpm<-function(counts,genelengths){
  common<-intersect(rownames(counts),names(genelengths))
  counts<-counts[common,]
  lengths<-genelengths[common]
  intermediate<-counts
  for(i in 1:nrow(counts)){
    intermediate[i,]<-1E3*counts[i,]/lengths[i]
  }
  tpms<-apply(intermediate,2,function(x){1E6*x/sum(x)})
  return(tpms)
}
fname<-"data/harenza/tpms.rda"
if(!file.exists(fname)){
  tpms<-tpm(rawcounts,genelengths)
  save(tpms,file=fname)
}

# Variance-Stabilizing Transformation (expmat)
fname<-"data/harenza/vstmat.rda"
if(!file.exists(fname)){
  vstmat<-vst(rawcounts)
  save(vstmat,file=fname)
}
```

## 5.2 Comparison between single cell and bulk RNA-Seq

```
### Load data ----
# Harenza
load("data/harenza/tpms.rda")
harenza<-tpms
# Our own
load("data/tpms.rda")
data<-tpms
bmat<-data[,grep("be2c",colnames(data))]
kmat<-data[,grep("kelly",colnames(data))]
b<-apply(bmat,1,sum)
k<-apply(kmat,1,sum)
# Correct names
colnames(harenza)[colnames(harenza)=="SK-N-BE-2--C"]<-"BE2C"
colnames(harenza)[colnames(harenza)=="SK-N-BE-2-"]<-"BE2"


### Correlation matrices ----
output<-matrix(NA,nrow=ncol(harenza),ncol=2)
colnames(output)<-c("SCC","SCC p-value")
rownames(output)<-colnames(harenza)

# Kelly
grpm<-k
for (i in 1:ncol(harenza)){
    cellname<-colnames(harenza)[i]
    message("Doing ",cellname)
    hrpm<-harenza[,cellname]
    common<-intersect(names(grpm),names(hrpm))
    grpm<-grpm[common]
    hrpm<-hrpm[common]
    x<-grpm
    y<-hrpm
    
    ii<-1
    for(cortype in c("s")){
        cortest<-cor.test(x,y,method=cortype)
        coeff<-signif(cortest$estimate,4)
        p<-signif(cortest$p.value,3)
        output[i,ii]<-coeff
        ii<-ii+1
        output[i,ii]<-p
        ii<-ii+1
    }
}
output<-output[order(-output[,1]),]
```

```
pander(output,style="rmarkdown") # Correlation between Kelly single cell dataset and other datasets
```

|  | SCC | SCC p-value |
| --- | --- | --- |
| **KELLY** | 0.9068 | 0 |
| **BE2C** | 0.8717 | 0 |
| **SK-N-DZ** | 0.8686 | 0 |
| **COG-N-519** | 0.8656 | 0 |
| **COG-N-415** | 0.8655 | 0 |
| **COG-N-453** | 0.8609 | 0 |
| **NB-69** | 0.8577 | 0 |
| **BE2** | 0.8571 | 0 |
| **NMB** | 0.8564 | 0 |
| **NBL-S** | 0.856 | 0 |
| **COG-N-561** | 0.8529 | 0 |
| **LA-N-5** | 0.8522 | 0 |
| **NGP** | 0.8507 | 0 |
| **NB-1643** | 0.8487 | 0 |
| **NB-EBc1** | 0.8481 | 0 |
| **COG-N-496** | 0.848 | 0 |
| **IMR-05** | 0.8479 | 0 |
| **COG-N-534** | 0.8473 | 0 |
| **CHP-134** | 0.8467 | 0 |
| **COG-N-471** | 0.8452 | 0 |
| **SK-N-SH** | 0.8441 | 0 |
| **SH-SY5Y** | 0.844 | 0 |
| **IMR-32** | 0.8435 | 0 |
| **SMS-KAN** | 0.8408 | 0 |
| **NB-SD** | 0.8404 | 0 |
| **NB-1691** | 0.8401 | 0 |
| **LA-N-6** | 0.8391 | 0 |
| **SMS-SAN** | 0.8384 | 0 |
| **NB-1** | 0.8382 | 0 |
| **COG-N-557** | 0.8373 | 0 |
| **SK-N-FI** | 0.8353 | 0 |
| **FELIX** | 0.8344 | 0 |
| **NLF** | 0.8344 | 0 |
| **COG-N-549** | 0.8311 | 0 |
| **SK-N-AS** | 0.8259 | 0 |
| **CHP-212** | 0.8222 | 0 |
| **NB-16** | 0.8045 | 0 |
| **RPE-1** | 0.7681 | 0 |
| **FETAL-BRAIN** | 0.7108 | 0 |
| **COG-N-440** | 0.2947 | 0 |

```
#png("plots/005_cortests_kelly_vs_harenza.png",w=2000,h=5000,res=350)
#grid.newpage()
#grid.table(output,theme=ttheme_default(base_colour="navy"))
#dev.off()
write.xlsx2(output,file="results/Supp_Table_S2_Kelly_vs_Bulk.xlsx")
```

```
# BE2C
output<-matrix(NA,nrow=ncol(harenza),ncol=2)
colnames(output)<-c("SCC","SCC p-value")
rownames(output)<-colnames(harenza)
grpm<-b
for (i in 1:ncol(harenza)){
    cellname<-colnames(harenza)[i]
    message("Doing ",cellname)
    hrpm<-harenza[,cellname]
    common<-intersect(names(grpm),names(hrpm))
    grpm<-grpm[common]
    hrpm<-hrpm[common]
    x<-grpm
    y<-hrpm
    
    ii<-1
    for(cortype in c("s")){
        cortest<-cor.test(x,y,method=cortype)
        coeff<-signif(cortest$estimate,4)
        p<-signif(cortest$p.value,3)
        output[i,ii]<-coeff
        ii<-ii+1
        output[i,ii]<-p
        ii<-ii+1
    }
}
output<-output[order(-output[,1]),]
```

```
pander(output,style="rmarkdown") # Correlation between BE2C single cell dataset and other datasets
```

|  | SCC | SCC p-value |
| --- | --- | --- |
| **BE2** | 0.8748 | 0 |
| **BE2C** | 0.872 | 0 |
| **COG-N-519** | 0.8583 | 0 |
| **COG-N-561** | 0.8557 | 0 |
| **NB-69** | 0.8534 | 0 |
| **COG-N-453** | 0.8473 | 0 |
| **KELLY** | 0.8472 | 0 |
| **NBL-S** | 0.8453 | 0 |
| **NB-1** | 0.845 | 0 |
| **NB-1643** | 0.8443 | 0 |
| **SK-N-FI** | 0.8441 | 0 |
| **COG-N-415** | 0.8431 | 0 |
| **SH-SY5Y** | 0.8424 | 0 |
| **SK-N-DZ** | 0.8424 | 0 |
| **LA-N-5** | 0.8421 | 0 |
| **NB-SD** | 0.8393 | 0 |
| **SK-N-AS** | 0.8387 | 0 |
| **SK-N-SH** | 0.8387 | 0 |
| **NMB** | 0.8359 | 0 |
| **NLF** | 0.8354 | 0 |
| **NB-EBc1** | 0.8353 | 0 |
| **FELIX** | 0.8335 | 0 |
| **CHP-212** | 0.8333 | 0 |
| **NB-1691** | 0.8325 | 0 |
| **SMS-SAN** | 0.832 | 0 |
| **CHP-134** | 0.831 | 0 |
| **NGP** | 0.831 | 0 |
| **COG-N-534** | 0.8305 | 0 |
| **COG-N-557** | 0.8304 | 0 |
| **SMS-KAN** | 0.8301 | 0 |
| **IMR-05** | 0.8282 | 0 |
| **LA-N-6** | 0.8262 | 0 |
| **COG-N-471** | 0.8244 | 0 |
| **IMR-32** | 0.8244 | 0 |
| **COG-N-496** | 0.8238 | 0 |
| **COG-N-549** | 0.8234 | 0 |
| **NB-16** | 0.8099 | 0 |
| **RPE-1** | 0.7782 | 0 |
| **FETAL-BRAIN** | 0.7082 | 0 |
| **COG-N-440** | 0.2951 | 0 |

```
#png("plots/005_cortests_be2c_vs_harenza.png",w=2000,h=5000,res=350)
#grid.table(output,theme=ttheme_default(base_colour="red3"))
#dev.off()
write.xlsx2(output,file="results/Supp_Table_S1_BE2C_vs_Bulk.xlsx")
```

```
### Scatter plots bulk vs sc ----
pseudo<-0.0001
toshow<-c("MYCN","GAPDH","GUSB","ACTB","B2M")
# Kelly
x<-log10(k+pseudo)
y<-log10(harenza[,"KELLY"]+pseudo)
#png("plots/005_scatter_kelly.png",w=4000,h=3000,res=600)
scatter(x,y,xlab="Single Cell RNA-Seq (Sum of TPMs)",ylab="Bulk RNA-Seq (TPM)",main="Kelly cells",col=col_kelly,method="spearman")
textplot3(x[toshow],y[toshow],words=toshow,font=2)
```

Figure S9. Scatter plot bulk vs sc Kelly

```
#dev.off()
```

```
# BE2C
x<-log10(b+pseudo)
y<-log10(harenza[,"BE2C"]+pseudo)

#png("plots/005_scatter_be2c.png",w=4000,h=3000,res=600)
scatter(x,y,xlab="Single Cell RNA-Seq (Sum of TPMs)",ylab="Bulk RNA-Seq (TPM)",main="BE2C cells",col=col_be2c,method="spearman")
textplot3(x[toshow],y[toshow],words=toshow,font=2)
```

Figure S10. Scatter plot bulk vs sc BE2C

```
#dev.off()
```

```
### Rtsne ----
# Load cell line annotation
cl<-read.csv("data/NBLcellLines.csv",header=TRUE)
cl<-setNames(cl[,4],cl[,1])

# Everything together
lh<-log10(harenza+pseudo)
lk<-log10(k+pseudo)
lb<-log10(b+pseudo)
common<-intersect(rownames(lh),intersect(names(lk),names(lb)))
tsnemat<-cbind(lh[common,],lk[common],lb[common])
colnames(tsnemat)[(ncol(tsnemat)-1):ncol(tsnemat)]<-c("scKELLY","scBE2C")
# Prepare the matrix
topvars<-names(sort(apply(tsnemat,1,var),decreasing=TRUE))[1:5000]
tsnemat<-tsnemat[topvars,]
# Seed for TSNE and calculate TSNE
set.seed(4)
ttt<-Rtsne(t(tsnemat),perplexity=10)


# Start preparing the plot
x<-setNames(ttt$Y[,1],colnames(tsnemat))
y<-setNames(ttt$Y[,2],colnames(tsnemat))
# Shapes
shapes<-rep(15,length(x))
names(shapes)<-names(x)
shapes[names(cl[cl=="notAmplified"])]<-16
#
#png("plots/005_tsne_with_bulk.png",w=3000,h=3000,res=500)
plot(x,y,pch=shapes,xlab="TSNE1",ylab="TSNE2",main="TSNE Representation of NBL Cell Lines",
     xlim=1.1*c(min(x),max(x)),ylim=1.1*c(min(y),max(y)),
     col=c(rep("#00000099",length(x)-2),col_kelly,col_be2c)
)
set.seed(4)
textplot3(x,y,words=names(x),cex=0.8,
          font=c(rep(1,length(x)-2),2,2),
          col=c(rep("black",length(x)-2),col_kelly,col_be2c),padding="  ",pos=3,offset=0.1
)
legend("bottomleft",pch=c(15,16),c("Amplified","Not Amplified"),title="MYCN status")
```

Figure S11. TSNE of bulk RNA-Seq NBL cell lines and single cell RNA-Seq

```
#dev.off()
```

```
# Again without BE2C
x<-setNames(ttt$Y[,1],colnames(tsnemat))
y<-setNames(ttt$Y[,2],colnames(tsnemat))
x<-x[1:(length(x)-1)]
y<-y[1:(length(y)-1)]
# Shapes
shapes<-rep(15,length(x))
names(shapes)<-names(x)
shapes[names(cl[cl=="notAmplified"])]<-16
#
#png("plots/005_tsne_with_bulk_Kelly.png",w=6000,h=3000,res=600)
plot(x,y,pch=shapes,xlab="TSNE1",ylab="TSNE2",main="Clustering of NBL Cell Lines",
     xlim=1.1*c(min(x),max(x)),ylim=1.1*c(min(y),max(y)),
     col=c(rep("#00000099",length(x)-1),col_kelly)
)
set.seed(4)
textplot3(x,y,words=names(x),cex=0.8,
          font=c(rep(1,length(x)-1),2),
          col=c(rep("black",length(x)-1),col_kelly),padding="  ",pos=3,offset=0.1
)
legend("bottomleft",pch=c(15,16),c("Amplified","Not Amplified"),title="MYCN status")
```

Figure S12. TSNE of bulk RNA-Seq NBL cell lines and single cell Kelly RNA-Seq

```
#dev.off()
```

# 6 Clustering single cell data

```
### Load Seurat object (already LogNormalized)
load("data/seuset.rda")
load("data/rawcounts_symbols.rda")


### Further processing ----
seuset<-FindVariableFeatures(seuset,selection.method="vst",nfeatures=Inf)
all.genes<-rownames(seuset)
seuset<-ScaleData(seuset,features=all.genes)
expmat<-as.matrix(seuset[["RNA"]]@scale.data)
dim(expmat) # 15782 genes, 2067 cells
```

```
## [1] 15782  2067
```

```
### Seurat-based clustering ----
PCA<-RunPCA(seuset,features=VariableFeatures(seuset))
```

```
# TSNE
set.seed(1)
TSNE<-RunTSNE(PCA)
#png("plots/006a_tsne_seurat_clustering.png",width=3000,height=3000, res=600)
DimPlot(TSNE,reduction="tsne",cols=c(col_be2c,col_kelly))+ggtitle('Seurat TSNE clustering of KELLY and BE2C')+theme(plot.title=element_text(hjust=0.5))
```

Figure S13. Seurat TSNE clustering of Kelly and BE2C

```
#dev.off()
```

```
# UMAP
set.seed(1)
UMAP<-RunUMAP(PCA, dims = 1:10)
#png("plots/006a_umap_seurat_clustering.png",width=3000,height=3000, res=600)
DimPlot(UMAP, reduction = 'umap',cols=c(col_be2c,col_kelly))+ggtitle('Seurat UMAP clustering of KELLY and BE2C')+  theme(plot.title = element_text(hjust = 0.5))
```

Figure S14. Seurat UMAP clustering of Kelly and BE2C

```
#dev.off()
```

## 6.1 Cell Cycle clustering

```
### Effects of cell cycle and read numbers on clustering ----
# Cell Cycle Markers, from Tirosh et al, 2015
ccgenes<-readLines("data/regev_lab_cell_cycle_genes.txt")
ccgenes<-eg2sym(sym2eg(ccgenes))
s.genes <- ccgenes[1:43]
g2m.genes <- ccgenes[44:97]
s.genes<-intersect(s.genes,rownames(seuset))
g2m.genes<-intersect(g2m.genes,rownames(seuset))
# Apply scoring
seuset<-CellCycleScoring(seuset,s.features=s.genes,g2m.features=g2m.genes,set.ident=TRUE)

# Rtsne, top var genes
allvars<-apply(expmat,1,var)
topvars<-names(sort(allvars,dec=TRUE))[1:1000]
topvarmat<-expmat[topvars,]
fname<-"data/rtsne.rda"
if(!file.exists(fname)){
    set.seed(1)
    rtsne<-Rtsne(t(topvarmat))
    save(rtsne,file=fname)
}else{load(fname)}
```

```
# Coloring cell lines
x<-setNames(rtsne$Y[,1],colnames(topvarmat))
y<-setNames(rtsne$Y[,2],colnames(topvarmat))
mycols<-rep("black",ncol(topvarmat))
mycols[grep("be2c",colnames(topvarmat))]<-col_be2c
mycols[grep("kelly",colnames(topvarmat))]<-col_kelly
#png("plots/006b_tsne_seurat_celllines.png",w=3000,h=3000,res=600)
plot(x,y,pch=20,xlab="TSNE1",ylab="TSNE2",main="TSNE clustering of KELLY and BE2C",col=mycols,xlim=c(min(x),max(x)*1.5))
mtext(paste0(length(x)," cells"),cex=0.8,font=2)
grid()
legend("bottomright",pch=19,col=c(col_be2c,col_kelly),legend=c("BE2C","Kelly"))
```

Figure S15. TSNE clustering of Kelly and BE2C

```
#dev.off()
```

```
# Color cell cycle phases
phases<-setNames(as.character(seuset@meta.data$Phase),rownames(seuset@meta.data))
mycols<-phases
mycols[phases=="G1"]<-"salmon"
mycols[phases=="S"]<-"cornflowerblue"
mycols[phases=="G2M"]<-"seagreen"
mycols<-mycols[colnames(topvarmat)]
#png("plots/006b_tsne_seurat_celllcycle.png",w=3000,h=3000,res=600)
plot(x,y,pch=20,xlab="TSNE1",ylab="TSNE2",main="TSNE clustering of KELLY and BE2C",col=mycols,xlim=c(min(x),max(x)*1.5))
mtext(paste0(length(x)," cells"),cex=0.8,font=2)
grid()
legend("bottomright",pch=19,col=c("salmon","cornflowerblue","seagreen"),legend=c("G1","S","G2M"))
```

Figure S16. Cell cycle phases assigned to cells

```
#dev.off()
```

```
# Barplot cell cycle distribution
kphases<-phases[grep("kelly",names(phases))]
bphases<-phases[grep("be2c",names(phases))]
ktab<-table(kphases)[c("G1","S","G2M")]
btab<-table(bphases)[c("G1","S","G2M")]

#png("plots/006b_phases_barplots.png",w=3000,h=3000,res=500)
par(mfrow=c(1,2))
max<-max(c(ktab,btab))*1.2
bp<-barplot(ktab,col=c("salmon","cornflowerblue","seagreen"),main="Kelly",ylim=c(0,max),ylab="nr. cells")
mtext(paste0(length(kphases)," cells"),cex=0.8)
perc<-round(100*ktab/length(kphases),2)
text(bp,ktab,labels=paste0(perc,"%"),pos=3,font=3)
bp<-barplot(btab,col=c("salmon","cornflowerblue","seagreen"),main="BE2C",ylim=c(0,max))
mtext(paste0(length(bphases)," cells"),cex=0.8)
perc<-round(100*btab/length(bphases),2)
text(bp,btab,labels=paste0(perc,"%"),pos=3,font=3)
```

Figure S17. Barplot showing cell cycle distributions

```
#dev.off()
par(mfrow=c(1,1))
```

## 6.2 Read number clustering

```
# Color nr. reads coloring
nreads<-apply(rawcounts,2,sum)
colfunc <- colorRampPalette(c("cornflowerblue","red3","orange"))
mycols<-colfunc(100)[as.numeric(cut(nreads,breaks=100))]
#png("plots/006b_tsne_seurat_nreads.png",w=3000,h=3000,res=600)
layout(matrix(1:2,ncol=2), width = c(3,1),height = c(1,1))
par(mar=c(5.1,4.1,4.1,0.1))
plot(x,y,pch=20,xlab="TSNE1",ylab="TSNE2",main="TSNE clustering",col=mycols,xlim=c(min(x),max(x)*1.5))
mtext(paste0(length(x)," cells"),cex=0.8,font=2)
grid()
par(mar=c(5.1,0.1,4.1,0.1))
plot(c(0,2),c(0,1),type='n',axes=F,xlab='',ylab='',main='Nr. Reads (k)')
legend_image<-as.raster(rev(matrix(colfunc(100), ncol=1)))
rasterImage(legend_image,0,0,1,1)
text(x=1.5,y=seq(0,1,l=5),labels=round(quantile(nreads/1E3)))
```

Figure S18. Cells colored by number of reads

```
#dev.off()
```

```
# Read counts distributions
#png("plots/006b_nreads_lineplots.png",w=3000,h=3000,res=600)
knreads<-nreads[grep("kelly",names(nreads))]
bnreads<-nreads[grep("be2c",names(nreads))]
plot(density(knreads),col=col_kelly,lwd=3,ylim=c(0,4e-5),main="Nr. reads/cell distribution")
lines(density(bnreads),col=col_be2c,lwd=3)
legend("topright",legend=c(
    paste0("Kelly (mean=",round(mean(knreads),2),")"),
    paste0("BE2C (mean=",round(mean(bnreads),2),")")
),lwd=3,col=c(col_kelly,col_be2c))
```

Figure S19. Read counts distributions

```
#dev.off()
```

```
### Regress out cell cycle and nUMI ----
fname<-"data/seuset_regressed.rda"
if(!file.exists(fname)){
    seuset<-ScaleData(object=seuset,vars.to.regress=c("nUMI","S.Score","G2M.Score"))
    save(seuset,file=fname)
}else{load(fname)}
regmat<-as.matrix(seuset[["RNA"]]@scale.data)
dim(regmat) # 15782 genes 2067 cells
```

```
## [1] 15782  2067
```

```
save(regmat,file="data/regmat.rda")
topvarmat<-regmat

# Rtsne, top var genes
allvars<-apply(topvarmat,1,var)
topvars<-names(sort(allvars,dec=TRUE))[1:1000]
topvarmat<-topvarmat[topvars,]
set.seed(1)
rtsne<-Rtsne(t(topvarmat))
save(rtsne,file=fname)
```

```
# Coloring cell lines
x<-setNames(rtsne$Y[,1],colnames(topvarmat))
y<-setNames(rtsne$Y[,2],colnames(topvarmat))
mycols<-rep("black",ncol(topvarmat))
mycols[grep("be2c",colnames(topvarmat))]<-col_be2c
mycols[grep("kelly",colnames(topvarmat))]<-col_kelly
#png("plots/006c_tsne_seurat_celllines_postregr.png",w=3000,h=3000,res=600)
plot(x,y,pch=20,xlab="TSNE1",ylab="TSNE2",main="Post-regression TSNE",col=mycols,xlim=c(min(x),max(x)*1.5))
mtext(paste0(length(x)," cells"),cex=0.8,font=2)
grid()
legend("bottomright",pch=19,col=c(col_be2c,col_kelly),legend=c("BE2C","Kelly"))
```

Figure S20. Seurat TSNE clustering of Kelly and BE2C, after regressing out number of reads (nUMI) and cell cycle

```
#dev.off()
```

```
# Color cell cycle phases
phases<-setNames(as.character(seuset@meta.data$Phase),rownames(seuset@meta.data))
mycols<-phases
mycols[phases=="G1"]<-"salmon"
mycols[phases=="S"]<-"cornflowerblue"
mycols[phases=="G2M"]<-"seagreen"
mycols<-mycols[colnames(topvarmat)]
#png("plots/006c_tsne_seurat_celllcycle_postregr.png",w=3000,h=3000,res=600)
plot(x,y,pch=20,xlab="TSNE1",ylab="TSNE2",main="Post-regression TSNE",col=mycols,xlim=c(min(x),max(x)*1.5))
mtext(paste0(length(x)," cells"),cex=0.8,font=2)
grid()
legend("bottomright",pch=19,col=c("salmon","cornflowerblue","seagreen"),legend=c("G1","S","G2M"))
```

Figure S21. Seurat TSNE clustering of Kelly and BE2C, after regressing out number of reads (nUMI) and cell cycle, coloring cell cycle phases

```
#dev.off()
```

```
# Color nr. reads coloring
nreads<-apply(rawcounts,2,sum)
colfunc <- colorRampPalette(c("cornflowerblue","red3","orange"))
mycols<-colfunc(100)[as.numeric(cut(nreads,breaks=100))]
#png("plots/006c_tsne_seurat_nreads_postregr.png",w=3000,h=3000,res=600)
layout(matrix(1:2,ncol=2), width = c(2,1),height = c(1,1))
plot(x,y,pch=20,xlab="TSNE1",ylab="TSNE2",main="Post-regression TSNE",col=mycols,xlim=c(min(x),max(x)*1.5))
mtext(paste0(length(x)," cells"),cex=0.8,font=2)
grid()
plot(c(0,2),c(0,1),type='n',axes=F,xlab='',ylab='',main='Nr. Reads (k)')
legend_image<-as.raster(rev(matrix(colfunc(100), ncol=1)))
rasterImage(legend_image,0,0,1,1)
text(x=1.5,y=seq(0,1,l=5),labels=round(quantile(nreads/1E3)))
```

Figure S22. Seurat TSNE clustering of Kelly and BE2C, after regressing out number of reads (nUMI) and cell cycle, coloring number of reads

```
#dev.off()
```

# 7 Differential expression analysis

We will perform a general differential expression analysis between BE2C and Kelly cells. ## Testing the effects of regression Two analyses are performed: using log-normalized data before and after cell cycle and nr. reads regression.

```
### Perform differential expression analysis
load("data/seuset_regressed.rda")
load("data/expmat.rda") # LogNormalized by Seurat
load("data/regmat.rda") # LogNormalized by Seurat + Regressed out cycle and nUMI
regmat<-regmat+abs(min(regmat))
# Function to calculate Differential expression with Wilcoxon tests
wexp<-function(matx,maty){
    columns<-c("log2fc","wstat","p","fdr")
    output<-matrix(NA,nrow=nrow(matx),ncol=length(columns))
    colnames(output)<-columns
    rownames(output)<-rownames(matx)
    pb<-txtProgressBar(0,nrow(matx),style=3)
    for(i in 1:nrow(matx)){
        x<-matx[i,]
        y<-maty[i,]
        l2fc<-log2(mean(x)/mean(y))
        wt<-wilcox.test(x,y)
        p<-wt$p.value
        if(p==0){p<-1e-301}
        stat<--log10(p)*sign(l2fc)
        output[i,]<-c(l2fc,stat,p,NA)
        setTxtProgressBar(pb,i)
    }
    output[,"fdr"]<-p.adjust(output[,"p"],method="BH")
    return(as.data.frame(output,stringsAsFactors=FALSE))
}

# be2c vs. kelly (regressed)
fname<-"results/007_res_be2c_vs_kelly.rda"
if(!file.exists(fname)){
    g1<-regmat[,grep("kelly",colnames(regmat))]
    g2<-regmat[,grep("be2c",colnames(regmat))]
    res<-wexp(g2,g1)
    res<-res[order(res[,"p"]),]
    save(res,file=fname)
    write.csv(res,file="results/007_res_be2c_vs_kelly.csv")
}else{load(fname)}


# be2c vs. kelly (not regressed)
fname<-"results/007_res_be2c_vs_kelly_notregressed.rda"
if(!file.exists(fname)){
    g1<-expmat[,grep("kelly",colnames(expmat))]
    g2<-expmat[,grep("be2c",colnames(expmat))]
    res<-wexp(g2,g1)
    res<-res[order(res[,"p"]),]
    save(res,file=fname)
    write.csv(res,file="results/007_res_be2c_vs_kelly_notregressed.csv")
}else{load(fname)}
```

```
### Compare signatures (regressed and not regressed) ----
load("results/007_res_be2c_vs_kelly.rda")
sig_reg<-setNames(res$log2fc,rownames(res))
load("results/007_res_be2c_vs_kelly_notregressed.rda")
sig_notreg<-setNames(res$log2fc,rownames(res))
sig_reg<-sig_reg[!is.infinite(sig_reg)]
sig_notreg<-sig_notreg[!is.infinite(sig_notreg)]

#png("plots/007_sigreg_vs_signotreg.png",w=3000,h=3000,res=600)
scatter(sig_reg,sig_notreg,xlab="Signature, regressed",ylab="Signature, not regressed",main="BE2C vs. Kelly Differential Expression")
```

Figure S23. Differential Expression Signatures compared, with and without cell cycle and nr. Reads regression. Signature is expressed as log2 fold change, with sign positive: upregulated in BE2C, and negative: upregulated in Kelly)

```
#dev.off()
```

## 7.1 Comparison with bulk signature

We will compare the overall, transcriptome-wide log fold change between BE2C and Kelly in single cell data (without regression) and bulk RNA-Seq (data from Harenza et al., 2017).

```
### Bulk signature (from Harenza) ----
# We must compare log2FC, since the bulk RNA-Seq has only a single sample per condition
load("data/harenza/vstmat.rda")
hsig<-log2(vstmat[,"SK-N-BE-2--C"]/vstmat[,"KELLY"])

load("data/expmat.rda")
min(expmat[expmat!=0]) # 0.05
```

```
## [1] 0.05749469
```

```
expmat<-expmat+0.01
scb<-apply(expmat[,grep("be2c",colnames(expmat))],1,mean)
sck<-apply(expmat[,grep("kelly",colnames(expmat))],1,mean)
scsig<-log2(scb/sck)

#png("plots/007_sc_vs_bulk.png",w=3000,h=3000,res=600)
scatter(hsig,scsig,xlab="log2FC, Bulk RNA-Seq",ylab="log2FC, Single Cell RNA-Seq",main="BE2C vs. Kelly Differential Expression")
```

Figure S24. Comparing bulk signature (x axis) and single cell signature (y-axis) of transcriptome comparison between BE2C and Kelly cells

```
#dev.off()
```

## 7.2 Visualization of differential expression by volcano plot.

Volcano plots show, for each gene, the sign of the differential expression on the x-axis, and the significance on the y-axis.

```
### Volcano plot ----
load("results/007_res_be2c_vs_kelly.rda")
x<-setNames(res$log2fc,rownames(res))
y<-setNames(-log10(res$fdr),rownames(res))


#png("plots/007_volcanoes.png",w=6000,h=3000,res=600)
par(mfrow=c(3,2))
pthrs<-c(2,5,10,20,50,100)
toshow<-c("RPS25","RPL27","MYCN")
for(pthr in pthrs){
    plot(x,y,pch=20,xlab="log2 Fold Change",ylab="-log10(FDR)",col="#00000011",main="BE2C vs. Kelly")
    up<-which(x>0&y>pthr)
    points(x[up],y[up],col="#FF000011",pch=20)
    dn<-which(x<0&y>pthr)
    points(x[dn],y[dn],col="#0000FF11",pch=20)
    mtext(paste0("Significant at FDR=10^-",pthr,": ",length(up),"(up), ",length(dn),"(down)"),cex=0.6)
    if(pthr==2){
        text(x[toshow],y[toshow],labels=toshow,font=2)
    }
}
```

Figure S25. Volcano plot of the differential (regressed) expression BE2C vs. Kelly cells at different significance thresholds

```
#dev.off()
```

# 8 Pathway enrichment analysis

We will perform pathway enrichment analysis via gene set enrichment analysis (GSEA) using the entire BE2C vs Kelly signature based on thousands of cells. Also, we will perform single cell GSEA (ssGSEA).

## 8.1 Whole-signature GSEA

The analysis will be performed using the GSEA function (as implemented in the *fgsea* and *corto* R packages) on the regressed signature vs. the MSIGDB database.

```
fname<-"data/mlist.rda"
if(!file.exists(fname)){
  mdf<-msigdbr(species="Homo sapiens") # Retrieve all human gene sets
  mlist<-mdf %>% split(x=.$gene_symbol,f=.$gs_name)
  save(mlist,file=fname)
}else{load(fname)}


### Get signature BE2C vs. Kelly ----
load("results/007_res_be2c_vs_kelly.rda")
signature<-setNames(-log10(res$p)*sign(res$log2fc),rownames(res))

### Check contrast orientation ----
signature[1:10]
```

```
##   COLEC11      TPM2   HTATIP2     CRIP2     RPS3A     RPS10     PITX1      RPL6 
##  307.3239  306.7425  306.3242  305.4707 -305.4114 -305.1677  304.1563 -303.2123 
##     C1QBP     RPL11 
## -302.0182 -301.0000
```

```
load("data/tpms.rda")
mean(tpms["RPS25",grep("be2c",colnames(tpms))])
```

```
## [1] 6870.798
```

```
mean(tpms["RPS25",grep("kelly",colnames(tpms))])
```

```
## [1] 2765.678
```

```
mean(tpms["RPL27",grep("be2c",colnames(tpms))])
```

```
## [1] 4782.861
```

```
mean(tpms["RPL27",grep("kelly",colnames(tpms))])
```

```
## [1] 8658.97
```

```
# Positive is, as planned, higher in BE2C


### Run GSEA (fgsea function) ----
fname<-"results/008_gsea.rda"
if(!file.exists(fname)){
    library(fgsea)
    gsea<-fgsea(pathways=mlist,stats=signature,nperm=1E6,minSize=4,maxSize=Inf,nproc=7)
    gsea<-gsea[order(gsea$pval),]
    gsea<-as.data.frame(gsea)
    save(gsea,file=fname)
    # Save as supp table
    write.csv(gsea,file="results/008_gsea_BE2C_vs_Kelly.csv")
}else{load(fname)}

### Run GSEA (corto function) ----
fname<-"results/008_gsea_corto.rda"
if(!file.exists(fname)){
    gsea<-matrix(nrow=length(mlist),ncol=3)
    rownames(gsea)<-names(mlist)
    colnames(gsea)<-c("ES","NES","p")
    set.seed(1)
    pb<-txtProgressBar(0,length(mlist),style=3)
    for(i in 1:length(mlist)){
        pname<-names(mlist)[i]
        pathway<-mlist[[pname]]
        if(length(intersect(names(signature),pathway))>1){
            obj<-gsea(signature,pathway,method="pareto")
            gsea[pname,]<-c(obj$es,obj$nes,obj$p)
        }else{
            gsea[pname,]<-c(0,0,1)
        }
        setTxtProgressBar(pb,i)
    }
    fdr<-p.adjust(gsea[,"p"],method="BH")
    gsea<-cbind(gsea,fdr)
    colnames(gsea)[ncol(gsea)]<-"fdr"
    save(gsea,file=fname)
    # Save as supp table
    write.csv(gsea,file="results/008_gsea_BE2C_vs_Kelly_corto.csv")
}else{load(fname)}
```

```
# All pathways
gsea<-as.data.frame(gsea)
gsea<-gsea[order(-abs(gsea$NES)),]


### Table of top pathways ----
top<-gsea[gsea$NES<0,][1:15,]
top<-rbind(top,gsea[gsea$NES>0,][15:1,])
top<-top[order(top$NES),]
toplot<-setNames(top$NES,rownames(top))
# Format
#names(toplot)<-gsub("GO_","",names(toplot))
names(toplot)<-gsub("_"," ",names(toplot))
names(toplot)<-str_to_title(names(toplot))
#png("plots/008_gsea_be2c_vs_kelly.png",w=6000,h=3000,res=500)
par(mar=c(4,1,3,1))
bp<-barplot(toplot,horiz=TRUE,xlab="Normalized Enrichment Score",
            xlim=1.3*c(-max(abs(toplot)),max(abs(toplot))),
            main="BE2C vs. Kelly, top Pathways",
            col=rep(c("cornflowerblue","salmon"),each=15),
            yaxt="n",cex.main=2
)
text(0,bp[1:15,1],names(toplot)[1:15],pos=4)
text(0,bp[16:30,1],names(toplot)[16:30],pos=2)
abline(v=c(-p2z(0.05),p2z(0.05)),lty=2)
```

Figure S26. Top significant pathways in the BE2C vs. Kelly comparison according to GSEA. Red bars shiow pathways upregulated in BE2C cells, blue bars in Kelly cells.

```
#dev.off()
```

We can also check for specific pathways.

```
### Check for Neuroblastoma ----
gsea[grep("NEUROBLASTOMA|NBL",rownames(gsea)),]
```

```
##                                                                                ES
## LASTOWSKA_NEUROBLASTOMA_COPY_NUMBER_UP                                 -0.3602327
## HEIDENBLAD_AMPLIFIED_IN_PANCREATIC_CANCER                              -0.4790722
## HEIDENBLAD_AMPLIFIED_IN_BONE_CANCER                                     0.9212140
## HEIDENBLAD_AMPLICON_8Q24_DN                                            -0.4671759
## LASTOWSKA_NEUROBLASTOMA_COPY_NUMBER_DN                                  0.3525325
## CHEN_NEUROBLASTOMA_COPY_NUMBER_GAINS                                   -0.4278639
## WHITE_NEUROBLASTOMA_WITH_1P36.3_DELETION                               -0.5554412
## OKAWA_NEUROBLASTOMA_1P36_31_DELETION                                   -0.5463855
## HEIDENBLAD_AMPLICON_12P11_12_UP                                         0.5054333
## ASGHARZADEH_NEUROBLASTOMA_POOR_SURVIVAL_DN                              0.4765018
## HEIDENBLAD_AMPLIFIED_IN_SOFT_TISSUE_CANCER                              0.5749628
## HEIDENBLAD_AMPLICON_12P11_12_DN                                         0.4690097
## HEIDENBLAD_AMPLICON_8Q24_UP                                             0.4333919
## REACTOME_UNBLOCKING_OF_NMDA_RECEPTORS_GLUTAMATE_BINDING_AND_ACTIVATION  0.3505902
##                                                                               NES
## LASTOWSKA_NEUROBLASTOMA_COPY_NUMBER_UP                                 -3.0902323
## HEIDENBLAD_AMPLIFIED_IN_PANCREATIC_CANCER                              -2.5426988
## HEIDENBLAD_AMPLIFIED_IN_BONE_CANCER                                     2.4164999
## HEIDENBLAD_AMPLICON_8Q24_DN                                            -2.1320833
## LASTOWSKA_NEUROBLASTOMA_COPY_NUMBER_DN                                  2.0976189
## CHEN_NEUROBLASTOMA_COPY_NUMBER_GAINS                                   -2.0857641
## WHITE_NEUROBLASTOMA_WITH_1P36.3_DELETION                               -2.0748547
## OKAWA_NEUROBLASTOMA_1P36_31_DELETION                                   -1.9188762
## HEIDENBLAD_AMPLICON_12P11_12_UP                                         1.5804668
## ASGHARZADEH_NEUROBLASTOMA_POOR_SURVIVAL_DN                              1.4502099
## HEIDENBLAD_AMPLIFIED_IN_SOFT_TISSUE_CANCER                              1.1406875
## HEIDENBLAD_AMPLICON_12P11_12_DN                                         1.1146510
## HEIDENBLAD_AMPLICON_8Q24_UP                                             1.0825685
## REACTOME_UNBLOCKING_OF_NMDA_RECEPTORS_GLUTAMATE_BINDING_AND_ACTIVATION  0.6037648
##                                                                                 p
## LASTOWSKA_NEUROBLASTOMA_COPY_NUMBER_UP                                 0.00200000
## HEIDENBLAD_AMPLIFIED_IN_PANCREATIC_CANCER                              0.01100000
## HEIDENBLAD_AMPLIFIED_IN_BONE_CANCER                                    0.01567053
## HEIDENBLAD_AMPLICON_8Q24_DN                                            0.03300000
## LASTOWSKA_NEUROBLASTOMA_COPY_NUMBER_DN                                 0.03593882
## CHEN_NEUROBLASTOMA_COPY_NUMBER_GAINS                                   0.03700000
## WHITE_NEUROBLASTOMA_WITH_1P36.3_DELETION                               0.03800000
## OKAWA_NEUROBLASTOMA_1P36_31_DELETION                                   0.05500000
## HEIDENBLAD_AMPLICON_12P11_12_UP                                        0.11400000
## ASGHARZADEH_NEUROBLASTOMA_POOR_SURVIVAL_DN                             0.14700000
## HEIDENBLAD_AMPLIFIED_IN_SOFT_TISSUE_CANCER                             0.25400000
## HEIDENBLAD_AMPLICON_12P11_12_DN                                        0.26500000
## HEIDENBLAD_AMPLICON_8Q24_UP                                            0.27900000
## REACTOME_UNBLOCKING_OF_NMDA_RECEPTORS_GLUTAMATE_BINDING_AND_ACTIVATION 0.54600000
##                                                                               fdr
## LASTOWSKA_NEUROBLASTOMA_COPY_NUMBER_UP                                 0.06913557
## HEIDENBLAD_AMPLIFIED_IN_PANCREATIC_CANCER                              0.13154998
## HEIDENBLAD_AMPLIFIED_IN_BONE_CANCER                                    0.15001402
## HEIDENBLAD_AMPLICON_8Q24_DN                                            0.20396252
## LASTOWSKA_NEUROBLASTOMA_COPY_NUMBER_DN                                 0.21110361
## CHEN_NEUROBLASTOMA_COPY_NUMBER_GAINS                                   0.21316641
## WHITE_NEUROBLASTOMA_WITH_1P36.3_DELETION                               0.21545338
## OKAWA_NEUROBLASTOMA_1P36_31_DELETION                                   0.24764922
## HEIDENBLAD_AMPLICON_12P11_12_UP                                        0.32511486
## ASGHARZADEH_NEUROBLASTOMA_POOR_SURVIVAL_DN                             0.35559789
## HEIDENBLAD_AMPLIFIED_IN_SOFT_TISSUE_CANCER                             0.43798740
## HEIDENBLAD_AMPLICON_12P11_12_DN                                        0.44557553
## HEIDENBLAD_AMPLICON_8Q24_UP                                            0.45392423
## REACTOME_UNBLOCKING_OF_NMDA_RECEPTORS_GLUTAMATE_BINDING_AND_ACTIVATION 0.62997551
```

```
### Plot top GSEAs ----
pathways<-c("REACTOME_METABOLISM_OF_RNA","CHICAS_RB1_TARGETS_CONFLUENT","GO_RIBOSOME_BIOGENESIS","LASTOWSKA_NEUROBLASTOMA_COPY_NUMBER_UP","LASTOWSKA_NEUROBLASTOMA_COPY_NUMBER_DN")
for(pathway in pathways){
    set<-mlist[[pathway]]
    obj<-gsea(signature,set,method="pareto")
    #png(paste0("plots/008_gsea_",pathway,".png"),w=3000,h=3000,res=600)
    plot_gsea(obj,ext_nes=gsea[rownames(gsea)==pathway,"NES"],title=pathway,colBarcode = "#00000033")
    #dev.off()
}
```

Figure S27. Specific pathways tested by GSEA in the BE2C vs Kelly single cell comparison

Figure S27. Specific pathways tested by GSEA in the BE2C vs Kelly single cell comparison

Figure S27. Specific pathways tested by GSEA in the BE2C vs Kelly single cell comparison

Figure S27. Specific pathways tested by GSEA in the BE2C vs Kelly single cell comparison

Figure S27. Specific pathways tested by GSEA in the BE2C vs Kelly single cell comparison

```
## Double gsea (TEST)
path1<-"LASTOWSKA_NEUROBLASTOMA_COPY_NUMBER_UP"
path2<-"LASTOWSKA_NEUROBLASTOMA_COPY_NUMBER_DN"
set1<-mlist[[path1]]
set2<-mlist[[path2]]
obj<-gsea2(signature,set1,set2,method="pareto")
plot_gsea2(obj)
```

Figure S27. Specific pathways tested by GSEA in the BE2C vs Kelly single cell comparison

## 8.2 Single-cell GSEA

```
load("data/seuset_regressed.rda")


### Calculate Rtsne ----
load("data/rtsne.rda")
load("data/regmat.rda")
x<-setNames(rtsne$Y[,1],colnames(regmat))
y<-setNames(rtsne$Y[,2],colnames(regmat))
```

```
### Overlay single genes over Rtsne ----
# Get TPM per cell
load("data/tpms.rda")
genes<-c("MYCN","GAPDH","ACTB","GUSB","B2M","ALK","LMO1")
for(gene in genes){
    vector<-rank(tpms[gene,])
    
    ## Color by TPM rank
    colfunc<-colorRampPalette(c("navy","grey","red3"))
    mycols<-colfunc(1000)[as.numeric(cut(vector,breaks=1000))]
    # Plot
    #png(paste0("plots/009_tsne_rank_",gene,".png"),w=4000,h=2000,res=450)
    layout(matrix(1:2,ncol=2), width = c(2,1),height = c(1,1))
    plot(x,y,pch=20,xlab="TSNE1",ylab="TSNE2",main=paste0(gene),col=mycols,xlim=c(min(x),max(x)*1.5))
    mtext(paste0(length(x)," cells"),cex=0.8,font=2)
    grid()
    plot(c(0,2),c(0,1),type='n',axes=F,xlab='',ylab='',main=paste0(" expression (rank)"))
    legend_image<-as.raster(rev(matrix(colfunc(1000), ncol=1)))
    rasterImage(legend_image,0,0,1,1)
    text(x=1.5,y=seq(0,1,l=5),labels=round(quantile(vector)))
    #dev.off()
}
```

Figure S28. Expression of selected genes in the single cell dataset

Figure S28. Expression of selected genes in the single cell dataset

Figure S28. Expression of selected genes in the single cell dataset

Figure S28. Expression of selected genes in the single cell dataset

Figure S28. Expression of selected genes in the single cell dataset

Figure S28. Expression of selected genes in the single cell dataset

Figure S28. Expression of selected genes in the single cell dataset

```
for(gene in genes){
    vector<-rank(tpms[gene,])
    
    ## Color by TPM rank
    colfunc<-colorRampPalette(c("navy","grey","red3"))
    mycols<-colfunc(1000)[as.numeric(cut(vector,breaks=1000))]

    ## Color by TPM
    colfunc<-colorRampPalette(c("navy","navy","navy","grey","salmon","red","red3"))
    mycols<-colfunc(1000)[as.numeric(cut(vector,breaks=1000))]
    vector<-tpms[gene,]
    pseudo<-min(vector[vector!=0]) # Pseudocount to ignore dropout effects
    vector<-log10(vector+pseudo)
    
    # Plot
    #png(paste0("plots/009_tsne_log10_",gene,".png"),w=4500,h=2000,res=500)
    lmatrix<-t(c(1,2,2,2,3))
    layout(lmatrix)
    par(mar=c(5.1,4.1,4.1,2.1))
    hist(vector,main=paste0(gene,""),xlab="Log10 TPM")
    mycols<-colfunc(1000)[as.numeric(cut(vector,breaks=1000))]
    plot(x,y,pch=20,xlab="TSNE1",ylab="TSNE2",main=paste0(gene),col=mycols,xlim=c(min(x),max(x)*1.5))
    mtext(paste0(length(x)," cells"),cex=0.8,font=2)
    par(mar=c(5.1,1.1,4.1,2.1))
    plot(c(0,2),c(0,1),type='n',axes=F,xlab='',ylab='',main=paste0("Log10 TPM"))
    legend_image<-as.raster(rev(matrix(colfunc(1000), ncol=1)))
    rasterImage(legend_image,0,0,1,1)
    text(x=1.5,y=seq(0,1,l=5),labels=round(seq(min(vector),max(vector),length.out=5),2))
    #dev.off()
}
```

Figure S29. Log 10 Expression of selected genes in the single cell dataset

Figure S29. Log 10 Expression of selected genes in the single cell dataset

Figure S29. Log 10 Expression of selected genes in the single cell dataset

Figure S29. Log 10 Expression of selected genes in the single cell dataset

Figure S29. Log 10 Expression of selected genes in the single cell dataset

Figure S29. Log 10 Expression of selected genes in the single cell dataset

Figure S29. Log 10 Expression of selected genes in the single cell dataset

```
### Load the msigdb database 7.1.1 ----
fname<-"data/mlist.rda"
if(!file.exists(fname)){
  mdf<-msigdbr(species="Homo sapiens") # Retrieve all human gene sets
  mlist<-mdf %>% split(x=.$gene_symbol,f=.$gs_name)
  save(mlist,file=fname)
}else{load(fname)}


### Calculate single-cell gsea (better to use area) ----
fname<-"results/009_ssgsea_regexpmat.rda"
load("data/regmat.rda") # LogNormalized by Seurat + Regressed out cycle and nUMI
# regmat<-regmat+abs(min(regmat))
if(!file.exists(fname)){
    scalemat<-t(scale(t(regmat)))
    ssgsea<-area(signatures=scalemat,groups=mlist,minsize=5)
    save(ssgsea,file=fname)    
}else{load(fname)}
```

```
### Extract highest significance + highest variance pathways ----
dim(ssgsea) # 24472 pathways, 2067 cells
```

```
## [1] 24472  2067
```

```
sumval<-apply(ssgsea,1,sum)
sumabs<-apply(ssgsea,1,function(x){sum(abs(x))})
var<-apply(ssgsea,1,var)

#png("plots/009b_ss_pathways.png",w=6000,h=2000,res=600)
par(mfrow=c(1,3))
plot(sumval,var,xlab="NES sum",ylab="NES variance")
plot(sumabs,var,xlab="NES sum of absolute",ylab="NES variance",main="Pathways in Single Cell Dataset")
toshow<-names(sort(var,dec=TRUE))[1:4]
text(sumabs[toshow],var[toshow],labels=toshow,pos=2,cex=0.4)
plot(sumabs,var/sumabs,xlab="NES sum of absolute",ylab="NES variance / sum of abs")
toshow<-names(sort(var/sumabs,dec=TRUE))[1:4]
text(sumabs[toshow],(var/sumabs)[toshow],labels=toshow,pos=2,cex=0.4)
```

Figure S30. Single Cell Pathway Enrichment Analysis: relationship between NES significance (sum) and NES variance

```
#dev.off()
```

```
### Highest variable within Kelly and BE2C ----
ssk<-ssgsea[,grep("kelly",colnames(ssgsea))]
ssb<-ssgsea[,grep("be2c",colnames(ssgsea))]
# Kelly
sumval<-apply(ssk,1,sum)
sumabs<-apply(ssk,1,function(x){sum(abs(x))})
var<-apply(ssk,1,var)
#png("plots/009b_ss_pathways_kelly.png",w=6000,h=2000,res=600)
par(mfrow=c(1,3))
plot(sumval,var,xlab="NES sum",ylab="NES variance")
plot(sumabs,var,xlab="NES sum of absolute",ylab="NES variance",main="Pathways in Kelly Cells")
toshow<-names(sort(var,dec=TRUE))[1:6]
text(sumabs[toshow],var[toshow],labels=toshow,pos=2,cex=0.4)
plot(sumabs,var/sumabs,xlab="NES sum of absolute",ylab="NES variance / sum of abs")
toshow<-names(sort(var/sumabs,dec=TRUE))[1:4]
text(sumabs[toshow],(var/sumabs)[toshow],labels=toshow,pos=2,cex=0.4)
```

Figure S31. Single Cell Pathway Enrichment Analysis: relationship between NES significance (sum) and NES variance in Kelly cells

```
#dev.off()
```

```
# BE2C
sumval<-apply(ssb,1,sum)
sumabs<-apply(ssb,1,function(x){sum(abs(x))})
var<-apply(ssb,1,var)
#png("plots/009b_ss_pathways_be2c.png",w=6000,h=2000,res=600)
par(mfrow=c(1,3))
plot(sumval,var,xlab="NES sum",ylab="NES variance")
plot(sumabs,var,xlab="NES sum of absolute",ylab="NES variance",main="Pathways in BE2C Cells")
toshow<-names(sort(var,dec=TRUE))[1:6]
text(sumabs[toshow],var[toshow],labels=toshow,pos=2,cex=0.4)
plot(sumabs,var/sumabs,xlab="NES sum of absolute",ylab="NES variance / sum of abs")
toshow<-names(sort(var/sumabs,dec=TRUE))[c(1,3,4)]
text(sumabs[toshow],(var/sumabs)[toshow],labels=toshow,pos=4,cex=0.4)
```

Figure S32. Single Cell Pathway Enrichment Analysis: relationship between NES significance (sum) and NES variance in BE2C cells

```
#dev.off()
```

```
### Overlay selected pathways over TSNE ----
colfunc<-colorRampPalette(c("navy","navy","navy","grey","salmon","red","red3"))
mycols<-colfunc(1000)[as.numeric(cut(vector,breaks=1000))]
pathways<-c("KEGG_RIBOSOME","MORF_SOD1","HALLMARK_MYC_TARGETS_V1","GO_OXIDATIVE_PHOSPHORYLATION")
for(pathway in pathways){
    vector<-ssgsea[pathway,]
    # Plot
    #png(paste0("plots/009b_ss_pathway_",pathway,".png"),w=4000,h=2000,res=450)
    layout(matrix(1:2,ncol=2), width = c(2,1),height = c(1,1))
    mycols<-colfunc(1000)[as.numeric(cut(vector,breaks=1000))]
    plot(x,y,pch=20,xlab="TSNE1",ylab="TSNE2",main=paste0(pathway),col=mycols,xlim=c(min(x),max(x)*1.5))
    mtext(paste0(length(x)," cells"),cex=0.8,font=2)
    plot(c(0,2),c(0,1),type='n',axes=F,xlab='',ylab='',main=paste0("Normalized Enrichment Score"),cex.main=0.8)
    legend_image<-as.raster(rev(matrix(colfunc(1000), ncol=1)))
    rasterImage(legend_image,0,0,1,1)
    text(x=1.5,y=seq(0,1,l=5),labels=round(seq(min(vector),max(vector),length.out=5),2))
    #dev.off()
}
```

Figure S33. Single Cell Pathway Enrichment Analysis: selected pathways

Figure S33. Single Cell Pathway Enrichment Analysis: selected pathways

Figure S33. Single Cell Pathway Enrichment Analysis: selected pathways

Figure S33. Single Cell Pathway Enrichment Analysis: selected pathways

```
### Bonus: correlation MYCN vs. MYC pathway ----
path<-ssgsea["HALLMARK_MYC_TARGETS_V1",]
gene<-tpms["MYCN",]
cols<-rep("white",ncol(tpms))
cols[grep("kelly",colnames(tpms))]<-col_kelly
cols[grep("be2c",colnames(tpms))]<-col_be2c
#png("plots/009c_MYCN_vs_MYCpathway.png",w=3000,h=3000,res=600)
scatter(path,gene,col=cols,xlab="MSIGDB Hallmark MYC V1 Targets (NES)",ylab="MYCN Expression (TPM)",
        main="MYCN vs. MYC pathway"
)
legend("topright",pch=19,col=c(col_be2c,col_kelly),legend=c("BE2C","Kelly"))
```

Figure S34. Correlation between MYCN expression and MSigDB MYC pathway activation

```
#dev.off()
```

# 9 Master Regulator Analysis

Master Regulator Analysis (MRA) focuses on identifying which transcription factors are the most likely controllers in regulating the observed transcriptional changes between two conditions. In our case, Kelly and BE2C cells. We will use here the MRA implemented by the R CRAN package *corto*, based on gene networks defined in Neuroblastoma datasets.

```
load("data/regmat.rda")
be2c<-regmat[,grep("be2c",colnames(regmat))]
kelly<-regmat[,grep("kelly",colnames(regmat))]

### Get regulons from independent datasets ----
tfs<-read.delim("code/tfgenes_2020_09_11.txt",header=FALSE)[,2]

# Generate networks for all NBL datasets
ds<-c("kocak_NBL","nrc_NBL","target_NBL")
for(d in ds){
    message("Doing ",d)
    fname<-paste0("networks/",d,"-regulon.rda")
    if(!file.exists(fname)){
        load(paste0("../masterset/data/",d,"-expmat.rda")) # This step requires the original data, not included here
        regulon<-corto(expmat,tfs,nbootstraps=100,p=1e-8,nthreads=7,verbose=TRUE)
        save(regulon,file=fname)
    }
}


### Run BE2C vs. Kelly MRA ----
fname<-"results/010_mra.rda"
if(!file.exists(fname)){
    # Run master regulator analysis (target dataset)
    load("networks/target_NBL-regulon.rda")
    corto_tmra<-mra(be2c,kelly,regulon=regulon,minsize=10,nthreads=8)
    # Run master regulator analysis (kocak dataset)
    load("networks/kocak_NBL-regulon.rda")
    corto_kmra<-mra(be2c,kelly,regulon=regulon,minsize=10,nthreads=8)
    # Run master regulator analysis (nrc dataset)
    load("networks/nrc_NBL-regulon.rda")
    corto_nmra<-mra(be2c,kelly,regulon=regulon,minsize=10,nthreads=8)
    #
    save(corto_tmra,corto_kmra,corto_nmra,file=fname)
}else{load(fname)}


### Agreement between MRAs ----
target<-corto_tmra$nes
kocak<-corto_kmra$nes
nrc<-corto_nmra$nes

# Significant MRs
sig_target_up<-names(which(corto_tmra$pvalue<=1e-180&corto_tmra$nes>0,useNames=TRUE))
sig_kocak_up<-names(which(corto_kmra$pvalue<=1e-180&corto_kmra$nes>0,useNames=TRUE))
sig_nrc_up<-names(which(corto_nmra$pvalue<=1e-180&corto_nmra$nes>0,useNames=TRUE))
length(sig_target_up) #
```

```
## [1] 58
```

```
length(sig_kocak_up) #
```

```
## [1] 62
```

```
length(sig_nrc_up) #
```

```
## [1] 89
```

```
sig_target_dn<-names(which(corto_tmra$pvalue<=1e-110&corto_tmra$nes<0,useNames=TRUE))
sig_kocak_dn<-names(which(corto_kmra$pvalue<=1e-110&corto_kmra$nes<0,useNames=TRUE))
sig_nrc_dn<-names(which(corto_nmra$pvalue<=1e-110&corto_nmra$nes<0,useNames=TRUE))
length(sig_target_dn) #
```

```
## [1] 50
```

```
length(sig_kocak_dn) #
```

```
## [1] 60
```

```
length(sig_nrc_dn) #
```

```
## [1] 39
```

```
# Intersection
int_up<-intersect(sig_target_up,intersect(sig_kocak_up,sig_nrc_up))
int_dn<-intersect(sig_target_dn,intersect(sig_kocak_dn,sig_nrc_dn))
length(int_up) #
```

```
## [1] 3
```

```
length(int_dn) #
```

```
## [1] 5
```

```
genes<-unique(c(int_up,int_dn))
length(genes) #
```

```
## [1] 8
```

```
# NOTCH1 is not a TF
genes<-setdiff(genes,"NOTCH1")
```

```
#png("plots/010_compareMRA.png",w=4000,h=2000,res=450)
set.seed(1) # for reproducible label placement
par(mfrow=c(1,2))
y<-spread.labs(kocak[genes],mindiff=4)
scatter(target,kocak,main="BE2C vs. Kelly Master Regulator Analysis",xlab="TARGET (NES)",ylab="Kocak (NES)",col="#00000099",xlim=c(-50,50),ylim=c(-50,60))
shadowtext(target[genes],y,labels=genes,col="white")
scatter(nrc,kocak,xlab="NRC (NES)",ylab="Kocak (NES)",col="#00000099",xlim=c(-50,50),ylim=c(-50,60))
shadowtext(nrc[genes],y,labels=genes,col="white")
```

Figure S35. Comparison between Master Regulator Analysis using two different Neuroblastoma network models (or “regulons”) inferred from the TARGET consoritum, Kocak et al. and NRC consortium

```
#dev.off()
```

```
#png("plots/010_mraplot_target.png",w=3000,h=8000,res=300)
mraplot(corto_tmra,mrs=genes)
```

Figure S36. Top Master Regulators from the TARGET dataset

```
#dev.off()
```

```
#png("plots/010_mraplot_kocak.png",w=3000,h=8000,res=300)
mraplot(corto_kmra,mrs=genes)
```

Figure S37. Top Master Regulators from the Kocak dataset

```
#dev.off()
```

```
#png("plots/010_mraplot_nrc.png",w=3000,h=8000,res=300)
mraplot(corto_nmra,mrs=genes)
```

Figure S38. Top Master Regulators from the NRC dataset

```
#dev.off()
```

# 10 Single Cell Master Regulator Analysis

```
load("data/regmat.rda")
load("results/010_mra.rda")
load(paste0("networks/target_NBL-regulon.rda"))
load("data/rtsne.rda")
x<-setNames(rtsne$Y[,1],colnames(regmat))
y<-setNames(rtsne$Y[,2],colnames(regmat))

### Single-cell master regulator analysis ----
fname<-"results/011_scmra.rda"
if(!file.exists(fname)){
    scmra<-mra(regmat,regulon=regulon)
    save(scmra,file=fname)
}else{load(fname)}
```

```
### Show top MRs ----
genes<-c("MYCN","DNAJC1","TWIST1","NOTCH1","E2F3","TEAD4")
for(gene in genes){
    vector<-scmra[gene,]
    
    ## Color by TPM
    colfunc<-colorRampPalette(c("navy","navy","navy","grey","salmon","red","red3"))
    mycols<-colfunc(1000)[as.numeric(cut(vector,breaks=1000))]
 
    # Plot
    png(paste0("plots/011_tsne_mra_",gene,".png"),w=4500,h=2000,res=500)
    lmatrix<-t(c(1,2,2,2,3))
    layout(lmatrix)
    par(mar=c(5.1,4.1,4.1,2.1))
    hist(vector,main=paste0(gene,""),xlab="NES")
    mycols<-colfunc(1000)[as.numeric(cut(vector,breaks=1000))]
    plot(x,y,pch=20,xlab="TSNE1",ylab="TSNE2",main=paste0(gene),col=mycols,xlim=c(min(x),max(x)*1.5))
    mtext(paste0(length(x)," cells"),cex=0.8,font=2)
    par(mar=c(5.1,1.1,4.1,2.1))
    plot(c(0,2),c(0,1),type='n',axes=F,xlab='',ylab='',main=paste0("NES"))
    legend_image<-as.raster(rev(matrix(colfunc(1000), ncol=1)))
    rasterImage(legend_image,0,0,1,1)
    text(x=1.5,y=seq(0,1,l=5),labels=round(seq(min(vector),max(vector),length.out=5),2))
    dev.off()
}
```

```
### Heatmap of single cell MR activity ----
source("../shared/functions/heatmaps.R")

## Select genes
# Manually selected
genes<-c("MYCN","E2F3","TEAD4","E2F1","MYC","E2F7")
# Most differentially active
newgenes<-c(
    "DNAJC1","ETV4","HEYL","HINFP","MBD3","NFRKB","NPAT","SCYL1","TAF10","TAF6","TWIST1","ZCCHC24","ZNF25","ZNHIT1",
    "SESN2","TRIM28","UXT","ZNF581"
)
genes<-c(genes,newgenes)
# Highest sum of absolute NES
sums<-sort(apply(scmra,1,function(x){sum(abs(x))}),dec=TRUE)
newgenes<-names(sums)[1:5] # "ZNF429" "RNF10"  "ZNF264" "ZBTB43" "MAX"
genes<-c(genes,newgenes)
#
genes<-unique(genes)

## Prepare table
toshow<-scmra[genes,]
toshow[toshow>5]<-5
toshow[toshow<(-5)]<--5
colside<-rep(col_be2c,ncol(scmra))
names(colside)<-colnames(scmra)
colside[grep("kelly",names(colside))]<-col_kelly

# Color function
colfun<-colorRampPalette(c("navy","navy","blue","blue","white","red","red","red3","red3"))


#png("plots/011_heatmap.png",w=4000,h=3000,res=300)
heatmap.3(toshow,mar=c(0,10),ColSideColors=t(t(colside)),KeyValueName="NES",col=colfun)
```

Figure S40. Mosti differentially active and varying Master Regulators in BE2C (salmon) and Kelly (blue) cells

```
#dev.off()
```

# 11 Louvain Clustering

The Louvain method for Community Detection is applied on the dataset to define clustering structure.

```
# Load Seurat object (already LogNormalized)
load("data/seuset.rda")
## Further processing
seuset<-FindVariableFeatures(seuset,selection.method="vst",nfeatures=Inf)
all.genes<-rownames(seuset)
seuset<-ScaleData(seuset,features=all.genes)
expmat<-as.matrix(seuset[["RNA"]]@scale.data)
dim(expmat) # 15782 genes, 2067 cells
```

```
## [1] 15782  2067
```

```
## PCA is performed considering most variable features, not the entire rows set
seuset<-RunPCA(seuset, features = VariableFeatures(object = seuset))
```

```
ElbowPlot(seuset) # choose the elbow, try +1 and -1
```

Figure S41. Elbow plot for the dataset Principal Components

This is defined by looking at the Elbow Plot above and ultimately keeping all Principal Components (PCs) that cumulatively explain at least 90% of the dataset variance.

```
## Determine the optimal number of significant PCs

## Determine the optimal number of significant PCs for subsequent clustering
## Determine percent of variation associated with each PC
pct<-seuset@reductions$pca@stdev / sum(seuset@reductions$pca@stdev) * 100
## Calculate cumulative percents for each PC
cum<-cumsum(pct)
## Determine which PC exhibits cumulative percent greater than 90% and % variation associated with the PC as less than 5
co1<-which(cum>90&pct<5)[1]
## Determine the difference between variation of PC and subsequent PC
co2 <- sort(which((pct[1:length(pct)-1] - pct[2:length(pct)]) > 0.1),  decreasing = T)[1] + 1 # last point where change of % of variation is more than 0.1%.
co2 # 13
```

```
## [1] 13
```

```
## Minimum of the two calculation
pcs <- min(co1, co2) # change to any other number
pcs # 13
```

```
## [1] 13
```

The number of PCs used for further visualization is `pcs`. We then run UMAP using the defined PCs.

```
seuset<- RunUMAP(seuset, dims = 1:pcs)
seuset<-FindNeighbors(object=seuset,dims=1:pcs,reduction='pca')
```

## 11.1 Assign cells to clusters

Cells are assigned to clusters using the Louvain method, at different resolution levels. ### Clusterint at Resolution=0.01 Defining two clusters: BE2C and Kelly cells.

```
seuset<-FindClusters(object=seuset,resolution=0.01)
```

```
## Modularity Optimizer version 1.3.0 by Ludo Waltman and Nees Jan van Eck
## 
## Number of nodes: 2067
## Number of edges: 69255
## 
## Running Louvain algorithm...
## Maximum modularity in 10 random starts: 0.9946
## Number of communities: 2
## Elapsed time: 0 seconds
```

```
table(seuset@meta.data$seurat_clusters)
```

```
## 
##    0    1 
## 1107  960
```

```
#png("plots/013b_umap_res001.png",w=1700,h=1500,res=300)
DimPlot(seuset,cols=c("cornflowerblue","salmon"))+ggtitle("Resolution=0.01")
```

Figure S42. Louvain Clustering at resolution=0.01 superimposed on UMAP coordinates

```
#dev.off()
```

Markers for each cluster can be then defined using Seurat’s methods. In this case, genes defining BE2C vs. Kelly cells (positive avg\_logFC specifies genes more upregulated in BE2C cells).

```
fname<-"results/013_markers_BE2C_vs_Kelly.rda"
if(!file.exists(fname)){
  markers<-FindMarkers(seuset,1,0) # min.pct=0.1, the default, will test only genes detected in at least 10% of either cell population
  save(markers,file=fname)
}else{load(fname)}
```

```
pander(markers[1:30,],style="rmarkdown")
```

|  | p\_val | avg\_logFC | pct.1 | pct.2 | p\_val\_adj |
| --- | --- | --- | --- | --- | --- |
| **LGALS1** | 0 | 2.318 | 0.997 | 0.467 | 0 |
| **VIM** | 0 | 2.234 | 0.999 | 0.557 | 0 |
| **S100A6** | 0 | 2.07 | 0.994 | 0.002 | 0 |
| **TMSB10** | 0 | 1.921 | 1 | 1 | 0 |
| **S100A11** | 0 | 1.892 | 0.994 | 0.032 | 0 |
| **VGF** | 0 | 1.855 | 0.964 | 0.228 | 0 |
| **SFRP1** | 0 | 1.732 | 0.999 | 0.715 | 0 |
| **PTN** | 0 | 1.6 | 0.99 | 0.194 | 0 |
| **ANXA2** | 0 | 1.559 | 0.999 | 0.501 | 0 |
| **RPS4Y1** | 0 | 1.482 | 0.974 | 0.003 | 0 |
| **COTL1** | 0 | 1.283 | 0.981 | 0.014 | 0 |
| **KCNH2** | 0 | 1.244 | 0.999 | 0.715 | 0 |
| **MZT2B** | 0 | 1.228 | 0.998 | 0.982 | 0 |
| **IGFBP5** | 0 | 1.157 | 0.94 | 0.171 | 0 |
| **MZT2A** | 0 | 1.085 | 0.998 | 0.784 | 0 |
| **COLEC11** | 0 | 1.044 | 0.997 | 0.746 | 0 |
| **COX3** | 0 | 0.9644 | 1 | 1 | 0 |
| **IFI16** | 0 | 0.8967 | 0.928 | 0.003 | 0 |
| **ATP5MG** | 0 | 0.8836 | 1 | 1 | 0 |
| **CD99** | 0 | 0.8834 | 0.961 | 0.002 | 0 |
| **GPX4** | 0 | 0.8732 | 0.998 | 0.987 | 0 |
| **MTRNR2L1** | 0 | 0.8655 | 0.939 | 0.045 | 0 |
| **GUK1** | 0 | 0.8593 | 0.997 | 1 | 0 |
| **COX2** | 0 | 0.8508 | 1 | 1 | 0 |
| **MAPKAPK2** | 0 | 0.8445 | 0.92 | 0.069 | 0 |
| **ATP5MPL** | 0 | 0.7947 | 0.999 | 0.998 | 0 |
| **RPS25** | 0 | 0.7929 | 1 | 1 | 0 |
| **NRGN** | 0 | 0.7346 | 0.921 | 0.068 | 0 |
| **GATA4** | 0 | 0.6896 | 0.885 | 0.007 | 0 |
| **LGALS3BP** | 0 | 0.6864 | 0.906 | 0.026 | 0 |

### 11.1.1 Clustering at higher resolution (0.2)

Defining three clusters: two for BE2C cells and one for Kelly cells.

```
seuset <- FindClusters(object = seuset, resolution = 0.2)
```

```
## Modularity Optimizer version 1.3.0 by Ludo Waltman and Nees Jan van Eck
## 
## Number of nodes: 2067
## Number of edges: 69255
## 
## Running Louvain algorithm...
## Maximum modularity in 10 random starts: 0.9134
## Number of communities: 3
## Elapsed time: 0 seconds
```

```
table(seuset@meta.data$seurat_clusters)
```

```
## 
##    0    1    2 
## 1107  523  437
```

```
#png("plots/013b_umap_res02.png",w=1700,h=1500,res=300)
DimPlot(seuset,cols=c("cornflowerblue","red","red3"))+ggtitle("Resolution=0.2")
```

Figure S43. Louvain Clustering at resolution=0.2 superimposed on UMAP coordinates

```
#dev.off()
```

We define now markers for BE2C cluster 2 vs. BE2C cluster 1 cells.

```
fname<-"results/013_markers_BE2C_2_vs_BE2C_1.rda"
if(!file.exists(fname)){
  markers<-FindMarkers(seuset,2,1)
  save(markers,file=fname)
}else{load(fname)}
```

```
pander(markers[1:30,],style="rmarkdown")
```

|  | p\_val | avg\_logFC | pct.1 | pct.2 | p\_val\_adj |
| --- | --- | --- | --- | --- | --- |
| **RPSA** | 1.301e-149 | -0.9266 | 0.998 | 1 | 2.053e-145 |
| **RPL35A** | 4.434e-123 | 0.4821 | 1 | 1 | 6.998e-119 |
| **VCAN** | 7.56e-123 | -0.7471 | 0.268 | 0.962 | 1.193e-118 |
| **RPL15** | 3.177e-116 | -0.5966 | 0.998 | 1 | 5.013e-112 |
| **RPL29** | 2.996e-115 | -0.4138 | 1 | 1 | 4.728e-111 |
| **TMA7** | 5.28e-111 | -0.5362 | 0.995 | 1 | 8.333e-107 |
| **SAMD11** | 2.403e-108 | -0.7264 | 0.805 | 0.99 | 3.793e-104 |
| **RPL11** | 1.11e-106 | -0.5394 | 1 | 1 | 1.752e-102 |
| **PPP1R14A** | 8.543e-105 | 0.8999 | 0.945 | 0.428 | 1.348e-100 |
| **MAGEA4** | 2.41e-104 | 0.5621 | 0.899 | 0.333 | 3.804e-100 |
| **RPL32** | 4.358e-102 | -0.44 | 1 | 1 | 6.878e-98 |
| **SRM** | 2.214e-101 | -0.5842 | 0.986 | 1 | 3.494e-97 |
| **RPL22** | 1.891e-97 | -0.4962 | 1 | 1 | 2.985e-93 |
| **CDKAL1** | 2.704e-96 | -0.643 | 0.412 | 0.933 | 4.267e-92 |
| **RPL14** | 2.261e-95 | -0.5241 | 1 | 1 | 3.568e-91 |
| **RPL38** | 2.682e-94 | 0.4375 | 1 | 1 | 4.233e-90 |
| **ENO1** | 6.247e-90 | -0.5381 | 0.998 | 1 | 9.859e-86 |
| **RPLP0** | 1.503e-89 | -0.3071 | 1 | 1 | 2.372e-85 |
| **TMEM98** | 1.622e-85 | 0.5432 | 0.892 | 0.474 | 2.559e-81 |
| **RPL26L1** | 2.011e-84 | 0.5033 | 0.984 | 0.95 | 3.173e-80 |
| **ARL4C** | 1.071e-82 | -0.5558 | 0.501 | 0.924 | 1.69e-78 |
| **DOK4** | 2.467e-82 | -0.5997 | 0.961 | 0.998 | 3.894e-78 |
| **BRK1** | 1.142e-80 | -0.4322 | 0.986 | 1 | 1.803e-76 |
| **GNB1** | 6.674e-80 | -0.4684 | 0.934 | 0.987 | 1.053e-75 |
| **CCDC80** | 2.154e-79 | -0.4814 | 0.217 | 0.82 | 3.4e-75 |
| **RPL22L1** | 1.852e-78 | 0.6814 | 0.989 | 0.954 | 2.923e-74 |
| **EMC6** | 5.066e-77 | 0.4846 | 0.984 | 0.904 | 7.995e-73 |
| **KCTD12** | 6.546e-76 | -0.4657 | 0.279 | 0.826 | 1.033e-71 |
| **GPX1** | 2.925e-74 | -0.4056 | 0.986 | 1 | 4.616e-70 |
| **YWHAE** | 4.36e-74 | 0.4513 | 1 | 0.992 | 6.882e-70 |

# 12 Dissection of heterogeneity

We used the f-scLVM method (implemented by the Bioconductor slalom package) and the pathway annaotation from MSigDb v7.2/WikiPathways to scout for annotated and not-annotated sources of heterogeneity within the dataset.

```
fname<-"results/014_fclsvm_model.rda"
if(!file.exists(fname)){
  # Construct a SingleCellExperiment object
  load("data/rawcounts_symbols.rda")
  dim(rawcounts) # 22135 2067
  log2counts<-log2(rawcounts+0.001)
  vars<-sort(apply(log2counts,1,var),dec=TRUE)[1:5000]
  log2counts<-log2counts[names(vars),]
  dim(log2counts) # 13265 2067
  sce<-SingleCellExperiment::SingleCellExperiment(assays=list(logcounts=log2counts))
  
  # We will supply f-scLVM with genesets in a GeneSetCollection object.
  # We will use a curated annotation as suggested in the f-scLCM vignette
  # The following file was downloaded from MSigDB on Jan 20, 2021
  gmtfile<-"data/c2.cp.wikipathways.v7.2.symbols.gmt"
  genesets<-GSEABase::getGmt(gmtfile)
  
  # Generate a f-scLVM model
  model<-newSlalomModel(sce,genesets)
  # 194 annotated factors retained;  393 annotated factors dropped.
  # 1616  genes retained for analysis.
  # Initialize it
  model<-initSlalom(model)
  # Train it
  model<-trainSlalom(model,nIterations=1000)
  save(model,file=fname)
} else {load(fname)}
```

```
## 194 annotated factors retained;  393 annotated factors dropped.
## 1616  genes retained for analysis.
## pre-training model for faster convergence
## iteration 0
## Model not converged after 50 iterations.
## iteration 0
## Model not converged after 50 iterations.
## iteration 0
## Switched off factor 148
## Switched off factor 107
## Switched off factor 197
## Switched off factor 76
## Switched off factor 27
## Switched off factor 172
## Switched off factor 138
## Switched off factor 43
## Switched off factor 67
## Switched off factor 186
## Switched off factor 178
## Switched off factor 105
## Switched off factor 49
## Switched off factor 191
## Switched off factor 81
## Switched off factor 29
## Switched off factor 98
## Switched off factor 73
## Switched off factor 72
## Switched off factor 46
## Switched off factor 164
## Switched off factor 57
## Switched off factor 153
## Switched off factor 181
## Switched off factor 125
## Switched off factor 140
## Switched off factor 161
## Switched off factor 86
## Switched off factor 111
## Switched off factor 68
## Switched off factor 173
## Switched off factor 158
## Switched off factor 44
## Switched off factor 53
## Switched off factor 157
## Switched off factor 187
## Switched off factor 117
## Switched off factor 22
## Switched off factor 192
## Switched off factor 144
## Switched off factor 165
## Switched off factor 115
## Switched off factor 196
## Switched off factor 15
## Switched off factor 97
## Switched off factor 180
## Switched off factor 142
## Switched off factor 104
## Switched off factor 134
## Switched off factor 149
## Switched off factor 130
## Switched off factor 122
## Switched off factor 47
## Switched off factor 12
## Switched off factor 194
## Switched off factor 183
## Switched off factor 168
## Switched off factor 23
## Switched off factor 88
## Switched off factor 92
## Switched off factor 101
## Switched off factor 42
## Switched off factor 61
## Switched off factor 71
## Switched off factor 167
## Switched off factor 90
## Switched off factor 39
## Switched off factor 112
## Switched off factor 152
## Switched off factor 32
## Switched off factor 99
## Switched off factor 40
## Switched off factor 118
## Switched off factor 137
## Switched off factor 175
## Switched off factor 124
## Switched off factor 156
## Switched off factor 54
## Switched off factor 38
## Switched off factor 26
## Switched off factor 190
## Switched off factor 96
## Switched off factor 128
## Switched off factor 135
## Switched off factor 56
## Switched off factor 151
## Switched off factor 114
## Switched off factor 59
## Switched off factor 188
## Switched off factor 169
## Switched off factor 62
## Switched off factor 159
## Switched off factor 116
## Switched off factor 34
## Switched off factor 11
## Switched off factor 41
## Switched off factor 123
## Switched off factor 13
## Switched off factor 84
## Switched off factor 160
## Switched off factor 119
## Switched off factor 79
## Switched off factor 176
## Switched off factor 60
## Switched off factor 93
## Switched off factor 198
## Switched off factor 109
## Switched off factor 179
## Switched off factor 65
## Switched off factor 177
## Switched off factor 132
## Switched off factor 16
## Switched off factor 48
## Switched off factor 170
## Switched off factor 174
## Switched off factor 36
## Switched off factor 30
## Switched off factor 94
## Switched off factor 51
## Switched off factor 89
## Switched off factor 82
## Switched off factor 171
## Switched off factor 113
## Switched off factor 33
## Switched off factor 63
## Switched off factor 102
## Switched off factor 31
## Switched off factor 14
## Switched off factor 133
## Switched off factor 100
## Switched off factor 145
## Switched off factor 129
## Switched off factor 103
## Switched off factor 70
## Switched off factor 166
## Switched off factor 189
## Switched off factor 21
## Switched off factor 25
## Switched off factor 139
## Switched off factor 91
## Switched off factor 141
## Switched off factor 195
## Switched off factor 78
## Switched off factor 154
## Switched off factor 45
## Switched off factor 185
## Switched off factor 120
## Switched off factor 66
## Switched off factor 87
## Switched off factor 193
## Switched off factor 83
## Switched off factor 75
## Switched off factor 108
## Switched off factor 85
## Switched off factor 24
## Switched off factor 163
## Switched off factor 64
## Switched off factor 162
## Switched off factor 126
## Switched off factor 182
## Switched off factor 37
## Switched off factor 6
## Switched off factor 95
## Switched off factor 80
## Switched off factor 106
## Switched off factor 19
## Switched off factor 17
## Switched off factor 10
## Switched off factor 35
## Switched off factor 7
## Switched off factor 20
## Switched off factor 52
## Switched off factor 18
## Switched off factor 147
## Switched off factor 131
## Switched off factor 58
## iteration 100
## Switched off factor 5
## iteration 200
## iteration 300
## iteration 400
## iteration 500
## iteration 600
## iteration 700
## iteration 800
## iteration 900
## iteration 1000
## Model not converged after 1000 iterations.
```

We will then investigate the results of the identified sources of heterogeneity

```
pander(slalom::topTerms(model),style="rmarkdown")
```

Table continues below


| term |
| --- |
| WP\_CHOLESTEROL\_METABOLISM\_INCLUDES\_BOTH\_BLOCH\_AND\_KANDUTSCHRUSSELL\_PATHWAYS |
| WP\_CELL\_CYCLE |
| WP\_SYNAPTIC\_VESICLE\_PATHWAY |
| WP\_REGULATION\_OF\_MICROTUBULE\_CYTOSKELETON |
| WP\_RETINOBLASTOMA\_GENE\_IN\_CANCER |
| WP\_DNA\_REPLICATION |
| WP\_AMINO\_ACID\_METABOLISM |
| WP\_GENOTOXICITY\_PATHWAY |
| WP\_PARKINUBIQUITIN\_PROTEASOMAL\_SYSTEM\_PATHWAY |
| WP\_METABOLIC\_REPROGRAMMING\_IN\_COLON\_CANCER |
| WP\_STEROL\_REGULATORY\_ELEMENTBINDING\_PROTEINS\_SREBP\_SIGNALLING |
| WP\_VITAMIN\_D\_RECEPTOR\_PATHWAY |
| WP\_DNA\_IRDAMAGE\_AND\_CELLULAR\_RESPONSE\_VIA\_ATR |
| WP\_MESODERMAL\_COMMITMENT\_PATHWAY |
| WP\_CILIARY\_LANDSCAPE |
| WP\_VEGFAVEGFR2\_SIGNALING\_PATHWAY |

| relevance | type | n\_prior | n\_gain | n\_loss |
| --- | --- | --- | --- | --- |
| 1.34 | annotated | 16 | 0 | 0 |
| 0.8336 | annotated | 55 | 1 | 0 |
| 0.5653 | annotated | 16 | 0 | 0 |
| 0.3831 | annotated | 26 | 0 | 0 |
| 0.3192 | annotated | 52 | 0 | 0 |
| 0.261 | annotated | 22 | 0 | 0 |
| 0.2393 | annotated | 34 | 0 | 0 |
| 0.2219 | annotated | 21 | 0 | 0 |
| 0.2176 | annotated | 22 | 0 | 0 |
| 0.2142 | annotated | 22 | 0 | 0 |
| 0.1964 | annotated | 34 | 0 | 0 |
| 0.1428 | annotated | 30 | 0 | 0 |
| 0.1373 | annotated | 50 | 0 | 0 |
| 0.11 | annotated | 45 | 0 | 0 |
| 0.09485 | annotated | 105 | 0 | 0 |
| 0.06547 | annotated | 157 | 0 | 0 |

```
plotRelevance(model)
```

Figure S44. Top pathways ranked by relevance in the f-scLVM model, showing gene set size and the number of active genes gained or lost by the pathway

```
plotTerms(model)
```

Figure S45. Graph showing the most relevant factors identified by the f-scLVM model, both annotated in Wiki Pathways (blue) or not annotated (red)

```
plotLoadings(model,"hidden02")
```

Figure S46. Most relevant genes in the hidden02 component, the highest identified source of variance in the dataset, according to f-scLVM

```
plotLoadings(model,"WP_CHOLESTEROL_METABOLISM_INCLUDES_BOTH_BLOCH_AND_KANDUTSCHRUSSELL_PATHWAYS")
```

Figure S47. Most relevant genes in the Cholesterol Metabolism term, according to f-scLVM

```
plotLoadings(model,"WP_CELL_CYCLE")
```

Figure S48. Most relevant genes in the Cell Cycle term, according to f-scLVM

# 13 Technical Session Info

The following code describes the R environment used to generate this document and will help making it fully reproducible should there be future updates in any of the packages.

```
sessionInfo()
```

```
## R version 4.0.3 (2020-10-10)
## Platform: x86_64-w64-mingw32/x64 (64-bit)
## Running under: Windows 10 x64 (build 19042)
## 
## Matrix products: default
## 
## locale:
## [1] LC_COLLATE=English_United States.1252 
## [2] LC_CTYPE=English_United States.1252   
## [3] LC_MONETARY=English_United States.1252
## [4] LC_NUMERIC=C                          
## [5] LC_TIME=English_United States.1252    
## 
## attached base packages:
##  [1] grid      parallel  stats4    stats     graphics  grDevices utils    
##  [8] datasets  methods   base     
## 
## other attached packages:
##  [1] wordcloud_2.6               RColorBrewer_1.1-2         
##  [3] gplots_3.1.1                xlsx_0.6.5                 
##  [5] forcats_0.5.0               purrr_0.3.4                
##  [7] readr_1.4.0                 tidyr_1.1.2                
##  [9] tibble_3.0.5                tidyverse_1.3.0            
## [11] TeachingDemos_2.12          stringr_1.4.0              
## [13] slalom_1.12.0               Seurat_3.2.3               
## [15] Rtsne_0.15                  pander_0.6.3               
## [17] org.Mm.eg.db_3.12.0         org.Hs.eg.db_3.12.0        
## [19] msigdbr_7.2.1               gridExtra_2.3              
## [21] ggplot2_3.3.3               dplyr_1.0.3                
## [23] DESeq2_1.30.0               SummarizedExperiment_1.20.0
## [25] MatrixGenerics_1.2.0        matrixStats_0.57.0         
## [27] GenomicRanges_1.42.0        GenomeInfoDb_1.26.2        
## [29] corto_1.1.3                 biomaRt_2.46.0             
## [31] AnnotationDbi_1.52.0        IRanges_2.24.0             
## [33] S4Vectors_0.28.0            Biobase_2.50.0             
## [35] BiocGenerics_0.36.0        
## 
## loaded via a namespace (and not attached):
##   [1] reticulate_1.18             tidyselect_1.1.0           
##   [3] RSQLite_2.2.2               htmlwidgets_1.5.3          
##   [5] BiocParallel_1.24.1         munsell_0.5.0              
##   [7] codetools_0.2-18            ica_1.0-2                  
##   [9] future_1.21.0               miniUI_0.1.1.1             
##  [11] withr_2.4.0                 colorspace_2.0-0           
##  [13] highr_0.8                   knitr_1.30                 
##  [15] rstudioapi_0.13             SingleCellExperiment_1.12.0
##  [17] ROCR_1.0-11                 tensor_1.5                 
##  [19] rJava_0.9-13                listenv_0.8.0              
##  [21] labeling_0.4.2              GenomeInfoDbData_1.2.4     
##  [23] polyclip_1.10-0             farver_2.0.3               
##  [25] bit64_4.0.5                 parallelly_1.23.0          
##  [27] vctrs_0.3.6                 generics_0.1.0             
##  [29] xfun_0.20                   BiocFileCache_1.14.0       
##  [31] R6_2.5.0                    rsvd_1.0.3                 
##  [33] locfit_1.5-9.4              bitops_1.0-6               
##  [35] spatstat.utils_1.20-2       DelayedArray_0.16.0        
##  [37] RcppArmadillo_0.10.1.2.2    assertthat_0.2.1           
##  [39] promises_1.1.1              scales_1.1.1               
##  [41] gtable_0.3.0                globals_0.14.0             
##  [43] goftest_1.2-2               rlang_0.4.10               
##  [45] genefilter_1.72.0           splines_4.0.3              
##  [47] lazyeval_0.2.2              broom_0.7.3                
##  [49] BiocManager_1.30.10         yaml_2.2.1                 
##  [51] reshape2_1.4.4              abind_1.4-5                
##  [53] modelr_0.1.8                backports_1.2.0            
##  [55] httpuv_1.5.5                tools_4.0.3                
##  [57] ellipsis_0.3.1              ggridges_0.5.3             
##  [59] Rcpp_1.0.5                  plyr_1.8.6                 
##  [61] progress_1.2.2              zlibbioc_1.36.0            
##  [63] RCurl_1.98-1.2              prettyunits_1.1.1          
##  [65] rpart_4.1-15                openssl_1.4.3              
##  [67] deldir_0.2-9                pbapply_1.4-3              
##  [69] cowplot_1.1.1               zoo_1.8-8                  
##  [71] haven_2.3.1                 ggrepel_0.9.1              
##  [73] cluster_2.1.0               fs_1.5.0                   
##  [75] magrittr_2.0.1              RSpectra_0.16-0            
##  [77] data.table_1.13.6           scattermore_0.7            
##  [79] reprex_0.3.0                lmtest_0.9-38              
##  [81] RANN_2.6.1                  fitdistrplus_1.1-3         
##  [83] xlsxjars_0.6.1              hms_1.0.0                  
##  [85] patchwork_1.1.1             mime_0.9                   
##  [87] evaluate_0.14               xtable_1.8-4               
##  [89] XML_3.99-0.5                readxl_1.3.1               
##  [91] compiler_4.0.3              KernSmooth_2.23-18         
##  [93] crayon_1.3.4                htmltools_0.5.0            
##  [95] mgcv_1.8-33                 later_1.1.0.1              
##  [97] geneplotter_1.68.0          lubridate_1.7.9.2          
##  [99] DBI_1.1.1                   dbplyr_2.0.0               
## [101] MASS_7.3-53                 rappdirs_0.3.1             
## [103] Matrix_1.2-18               cli_2.2.0                  
## [105] igraph_1.2.6                pkgconfig_2.0.3            
## [107] plotly_4.9.3                xml2_1.3.2                 
## [109] annotate_1.68.0             XVector_0.30.0             
## [111] BH_1.75.0-0                 rvest_0.3.6                
## [113] digest_0.6.27               sctransform_0.3.2          
## [115] RcppAnnoy_0.0.18            graph_1.68.0               
## [117] spatstat.data_1.7-0         cellranger_1.1.0           
## [119] rmarkdown_2.6               leiden_0.3.6               
## [121] uwot_0.1.10                 GSEABase_1.52.1            
## [123] curl_4.3                    shiny_1.5.0                
## [125] gtools_3.8.2                lifecycle_0.2.0            
## [127] nlme_3.1-151                jsonlite_1.7.2             
## [129] fansi_0.4.2                 viridisLite_0.3.0          
## [131] askpass_1.1                 pillar_1.4.7               
## [133] lattice_0.20-41             fastmap_1.0.1              
## [135] httr_1.4.2                  plotrix_3.7-8              
## [137] survival_3.2-7              glue_1.4.2                 
## [139] spatstat_1.64-1             png_0.1-7                  
## [141] bit_4.0.4                   stringi_1.5.3              
## [143] blob_1.2.1                  caTools_1.18.1             
## [145] memoise_1.1.0               irlba_2.3.3                
## [147] future.apply_1.7.0
```
